# Twin Proliferation and Prolongation under Kinetic Control: Pd–Au Janus Icosahedra *versus* Pd@Au Core–Shell Starfishes

**DOI:** 10.1021/jacs.3c03682

**Published:** 2023-06-09

**Authors:** Xiaoyu Qiu, Veronica Pawlik, Shan Zhou, Jing Tao, Younan Xia

**Affiliations:** † The Wallace H. Coulter Department of Biomedical Engineering, Georgia Institute of Technology and Emory University, Atlanta, Georgia 30332, United States; ‡ School of Chemistry and Materials Science, Nanjing Normal University, Nanjing 210023, P. R. China; § School of Chemistry and Biochemistry, Georgia Institute of Technology, Atlanta, Georgia 30332, United States; ∥ Condensed Matter Physics and Materials Science Department, Brookhaven National Laboratory, Upton, New York 11973, United States

## Abstract

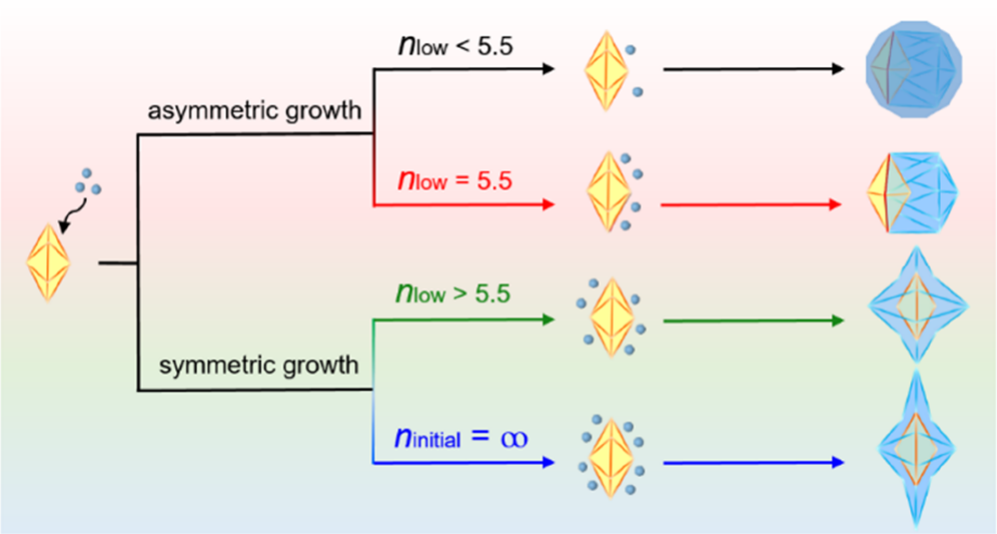

Heterogeneous bimetallic nanocrystals featuring explicit spatial configurations and abundant twin defects can simultaneously enable geometric and ligand effects to enhance catalytic and photonic applications. Herein, we report two growth patterns of Au atoms on penta-twinned Pd decahedra, involving twin proliferation to generate asymmetric Pd–Au Janus icosahedra and twin elongation to produce anisotropic Pd@Au core–shell starfishes, respectively. Mechanistic analysis indicates that the injection rate determines the lower-limit number (*n*_low_) of Au(III) ions in the steady state and thus controls the growth pattern. When *n*_low_ ≤ 5.5, the kinetic rate is slow enough to initiate asymmetrical one-side growth but fast enough to outpace surface diffusion; Au tetrahedral subunits are successively proliferated along the axial ⟨110⟩ direction of Pd decahedra to form Pd–Au Janus icosahedra. Composed of five Pd and 15 Au tetrahedral subunits, such a heterogeneous icosahedron supports high (2.2 GPa) tensile strain and high strain difference up to +21.9%. In contrast, when *n*_low_ > 5.5, the fast reduction kinetics promotes symmetric growth with inadequate surface diffusion. As such, Au atoms are laterally deposited along five high-indexed ⟨211⟩ ridges of Pd decahedra to generate concave Pd@Au core–shell starfishes with tunable sizes (28–40 nm), twin elongation ratios (33.82–162.08%), and lattice expansion ratios (8.82–20.10%).

## Introduction

Multitwinned nanocrystals with a high density of twin defects have received considerable attention in applications related to catalysis, photocatalysis, plasmon-enhanced spectroscopy, and biomedicine.^1,2^ The presence of twin boundaries can induce strong surface lattice strain and thus contribute to high mechanical strength, superior thermal stability, and enhanced catalytic activity.^3,4^ Notable examples include decahedral and icosahedral nanocrystals composed of 10 and 20, respectively, single-crystal tetrahedral subunits sharing both apexes and facets. On the other hand, heterogeneous bimetallic nanocrystals with specific spatial distributions of constituent elements are fascinating materials owing to the possible synergy arising from geometric effects (*e.g.*, lattice strain) and electronic interactions.^5,6^ For example, Janus structures characterized by the side-by-side connection of two metals can generate strong interfacial effects.^7^ For anisotropic core–shell structures, the shell metal offers more tunability in terms of shape, curvature, and aspect ratio than the isotropic counterpart.^8,9^ Taken together, considering the merits of twin defects and elemental distribution, constructing heterogeneous twin structures can generate the effects of both lattice strain and bimetallic reciprocity, holding the promise to significantly enhance the catalytic activity.

Using well-defined, multitwinned nanocrystals as seeds in the setting of seed-mediated growth has been identified as an effective method to construct heterogeneous bimetallic twin structures with inherited twin boundaries.^10,11^ Taking Pd decahedral seeds as an example, the growth of the second metal (*e.g.*, Au or Pt) can be divided into either asymmetric or symmetric mode, which can be further subdivided into axial and equatorial growth.^12^ At a slow reduction rate, nucleation of the second metal only occurs on a limited number of equivalent sites on the decahedral seeds, resulting in asymmetric growth.^13^ The asymmetric structures include axial growth-induced nanorods, nanopyramids, nanopencils, nanowires, and equatorial growth-induced lateral nanopyramids.^14,15^ At a fast reduction rate, in contrast, nucleation of the second metal tends to occur from all available sites on the decahedral seeds, leading to symmetric growth.^16,17^ Such symmetric growth can generate axial structures such as bidirectional pyramids and rods, or equatorial structures including core–shell/frame decahedra and core–shell starfishes.^18,19^

In most cases, the resultant heterogeneous metal-based decahedral derivatives can only replicate the original twin boundaries in the seed, giving the final nanocrystal a similar quantity and length or even covering the twin regions to make them disappear at the distal ends. The continuous proliferation and prolongation of pre-existing penta-twinned boundaries are greatly restricted by the high internal strain and surface free energies at the twin regions that are not favored by thermodynamics.^20^ Moreover, newly formed twin defects are highly active toward oxidation, making it difficult to preserve them on the surface.^21^ To date, we still lack effective methods for proliferating and/or elongating the twin boundaries in a controllable manner. Furthermore, it is still unclear about the mechanisms for twin proliferation and/or prolongation between heterogeneous metals, not mentioning their effects on the lattice strain.

Herein, we report a study of the kinetically controlled growth (asymmetric and axial versus symmetric and lateral) of Au atoms on Pd decahedral seeds, together with a systematic analysis of the growth mechanisms, strain distributions, and overgrowth behaviors. By simply adjusting the injection rate of the precursor, we could deterministically achieve twin proliferation to form asymmetric Pd–Au Janus icosahedra and twin elongation to produce anisotropic Pd@Au core–shell starfishes. Specifically, at a slow injection rate, the low concentration of newly formed Au atoms was restricted to one side of the Pd decahedral seed. This induced and sustained the asymmetric growth mode with a slow kinetic rate to transform a Pd decahedron into a Pd–Au Janus icosahedron, with 5 tetrahedral subunits composed of Pd and the remaining 15 composed of Au. Such a heterometallic configuration with an icosahedral shape exhibited a particular strain distribution and high strain difference (+21.9%). Furthermore, by reducing the number of Pd seeds, the overgrowth behavior of the Au atoms followed a sequentially asymmetric-conformal growth, generating Pd@Au core–shell icosahedra with twin elongation ratio (*R*_e_) up to 69.05%. In contrast, one-shot addition led to a high initial concentration of Au atoms for them to deposit on all available sites of the Pd decahedral seed at a fast kinetic rate. The preferential atomic deposition at the vertices, the major diffusion along the ridges relative to the edges, and the inadequate surface diffusion along the {111} faces gave rise to lateral growth along the 5-fold twin boundaries, generating anisotropic Pd@Au core–shell starfishes with *R*_e_ of 33.82% and a lattice expansion ratio of 8.82%.

## Experimental Section

### Chemicals and Materials

Sodium tetrachloropalladate(II) (Na_2_PdCl_4_, 99.998%), sodium sulfate (Na_2_SO_4_, 99.0%), tetrachloroauric(III) acid (HAuCl_4_·3H_2_O, ≥99.9%), silver nitrate (AgNO_3_, 99.8%), diethylene glycol (DEG, 99.0%, lot no. BCBJ9535), L-ascorbic acid (AA), poly(vinyl pyrrolidone) (PVP, MW ≈ 55,000), and cetyltrimethylammonium bromide (CTAB) were purchased from Sigma-Aldrich and used as received. The commercial Pd/C catalyst (20 wt %) was obtained from Johnson Matthey Chemicals Ltd. Deionized (DI) water of 18.2 MΩ cm in resistivity was used throughout the experiments.

### Instrumentation

Transmission electron microscopy (TEM) analysis was conducted using a Hitachi HT7700 (Tokyo, Japan) operated at 120 kV. High-resolution high-angle annular dark-field scanning transmission electron microscopy (HAADF-STEM) and tomography images were taken on a JEOL JEM 2200FS STEM/TEM microscope equipped with a CEOS probe corrector (Heidelberg, Germany) to provide a nominal image resolution of 0.07 nm. Energy dispersive X-ray (EDX) analysis was performed in STEM mode using an aberration-corrected JEOL 2200FS electron microscope equipped with a Bruker-AXS SDD detector. The metal contents were determined using an inductively coupled plasma mass spectrometer (ICP-MS, NexION 300Q, PerkinElmer). UV–vis-NIR extinction spectra were recorded on a Lambda 750 spectrometer (PerkinElmer).

### Synthesis of Pd–Au Janus Icosahedra and Pd@Au Core–Shell Starfishes

First, Pd decahedral seeds of 17 nm in size were synthesized using a protocol reported previously.^22^ The as-prepared Pd seeds were dispersed in DI water at a concentration of 1.0 mg mL^–1^ for further use.

For the synthesis of Pd–Au Janus icosahedra, 30 μL of the Pd decahedral seeds, 2 mL of CTAB (200 mM), 10 μL of AgNO_3_ (40 mM), and 40 μL of AA (100 mM) were mixed in a 20 mL vial and preheated in a thermostatic bath held at 37 °C for 5 min under magnetic stirring. Then, 2.0 mL of HAuCl_4_ (0.88 mM) solution was injected using a syringe pump (2.0 mL h^–1^). Each drop of the precursor solution was set to a volume of 0.002 mL. After injection, the reaction was allowed to continue for another 5 min. The solid products were collected by centrifugation at 13,200 rpm for 10 min and washed twice with water to obtain Pd–Au Janus icosahedra.

The synthesis of Pd@Au core–shell starfishes was identical to the procedure described above except for the injection rate. Instead of using a syringe pump, 2.0 mL of HAuCl_4_ (0.3 mg mL^–1^) was quickly added into the preheated mixture in one shot using a pipette. After the reaction proceeded for 15 min, the solid products were collected by centrifugation at 13,200 for 10 min and washed twice with water to obtain Pd@Au core–shell starfishes.

### Analysis of the Correlation between Growth Mode and Injection Rate

For dropwise injection, after the introduction of the first few drops, the instantaneous number (*n*_t_) of Au(III) precursor ions in the reaction mixture will quickly reach a steady state.^23^ Assuming that the reduction rate is independent of the initial precursor concentration, the consumption of the precursor from each drop can be derived separately. This means that *n_t_* can be expressed mathematically according to the following equation
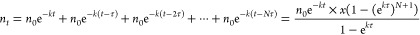
1Herein, *n*_0_ is the number of precursor ions in each drop; τ is the duration of time between adjacent drops; *N* is the total number of drops; and *k* is the reduction rate constant derived from ICP-MS analysis. In principle, any pair of *n*_0_ and τ can be used to calculate the *n*_*t*_. In this case, after a few drops, *n*_*t*_ will reach a steady state that fluctuates between a lower limit (*n*_low_) and an upper limit (*n*_up_). The values of *n*_up_ and *n*_low_ can be calculated according to the following equations:
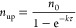
2
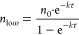
3In this work, we only adjust τ to tune the injection rate while keeping *n*_0_ fixed at all injection rates. Accordingly, when calculating the *n*_up_ and *n*_low_ at different injection rates, we use the same value for *n*_0_, making it a constant for our calculations. For simplicity, the value of *n*_0_ was assumed to be the same as the number of seed particles in the growth solution. To determine the rate constant (*k*), the concentrations of Au(III) ions at different time points of the synthesis were measured. These concentrations were used to perform a linear regression fit of the plot of ln[Au(III)] *versus* reaction time.^24^ Since the dosage of the reducing agent in this solution was in great excess relative to the precursor, a pseudo-first-order rate law could be assumed and expressed as
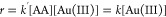
4The concentrations of unreacted Au(III) ions remaining in the reaction solution at different time points were analyzed using an ICP-MS. To do this, the reaction was run as described above. 2.0 mL of HAuCl_4_ (0.88 mM) was injected in one shot. As the reaction had proceeded for a certain period of time, 0.1 mL of aliquot was removed and immediately injected into 0.9 mL of a supersaturated KBr solution to quench the reduction. The solution was then centrifuged at 55,000 rpm for 10 min to precipitate out the Au nanocrystals, leaving behind residual Au(III) ions in the supernatant, which was then diluted with aqueous HNO_3_ solution for ICP-MS analysis.

### Molecular Dynamics (MD) Simulations

MD simulations were performed using the open-source Large-Scale Atomic/Molecular Massively Parallel Simulator (LAMMPS) code with an integration time step of 2 fs.^25^ The size of the Pd–Au Janus icosahedron was about 9 × 9 × 9 nm^3^ containing 23,822 atoms, whereas the size of the Pd@Au core–shell icosahedron was about 11 × 11 × 11 nm^3^ containing 45,418 atoms. Both models involved the same Pd decahedral seed containing 6,670 atoms. The embedded atom method (EAM) potential was adopted to analyze the FCC Au–Au and FCC Pd–Pd interactions, respectively. The interaction between Au and Pd atoms was analyzed using a Lennard-Jones (LJ) interatomic potential, in which the cutoff distance was 12 Å, the distance constant was 2.7582 Å, and the energy constant was 0.0018762 eV. During relaxation, the conjugate gradient method was applied to avoid overlaps in the positions of atoms and thus determine the stable configuration of models. Then, the models were equilibrated to 10 K using the canonical ensemble (NVT, constant volume and temperature) for 150 ps.

## Results And Discussion

### Deterministic Syntheses of Pd–Au Janus Icosahedra and Pd@Au Core–Shell Starfishes

**Figure 1:**
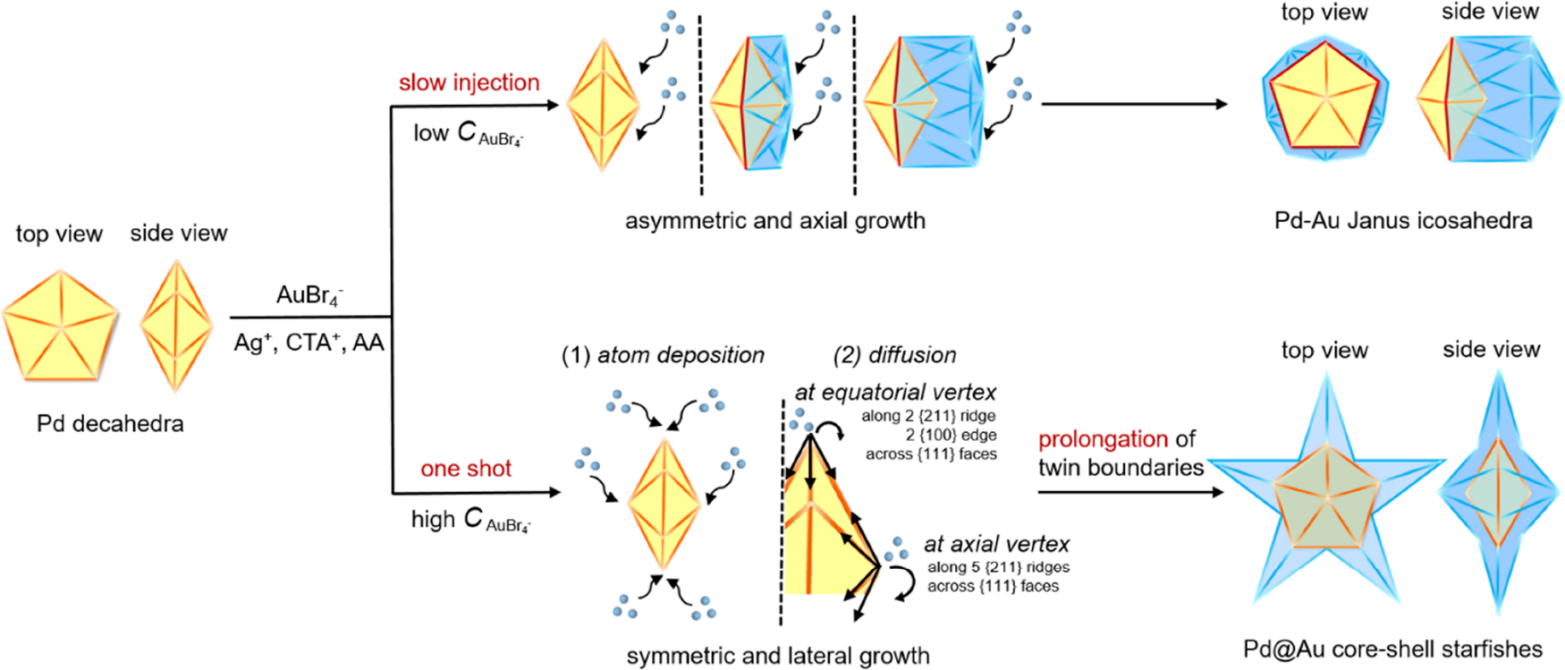
Schematic illustration of two different pathways that lead to the generation of Pd–Au Janus icosahedra and Pd@Au core–shell starfishes, respectively.

Figure 1 schematically illustrates two distinct pathways that generate Pd–Au Janus icosahedra and Pd@Au core–shell starfishes when the Au(III) precursor is added dropwise and in one shot, respectively. The penta-twinned Pd decahedral seeds were synthesized with a size of 17 nm (Figure S1) by following a previously reported protocol.^22^Figure S2 shows schematics of the top views, side views, and size definitions of a decahedral seed and its derivatives. As shown by the structural analysis in Figure S3, each decahedron contains ten {111} side faces, ten {211} ridges, and five {100} edges, as well as two different types of vertices. The energies of different surface sites are expected to decrease in the order of equatorial vertices > axial vertices > edges ≈ ridges > {111} faces.^26,27^

The merits of using Au to investigate the twin proliferation and prolongation on Pd decahedral seeds are as follows: (i) compared to Pd and Pt, Au is more inclined to form multiple twin boundaries because of its lower internal strain energy;^28^ (ii) Au endows strong chemical stability and weak oxygen bonding, helping protect the newly created twin defects from oxidative etching;^29^ and (iii) the large (4.8%) mismatch in lattice constant between Au and Pd is favorable for asymmetric growth of anisotropic structures. In a typical synthesis, the seed-mediated growth of Au was conducted at 37 °C in a mixture containing Pd decahedral seeds, HAuCl_4_ (precursor), AA (reducing agent), as well as AgNO_3_ (passivator) and CTAB. When AuCl_4_^–^ and CTAB were mixed in solution, a deep brown color was generated, suggesting the formation of AuBr_4_^–^ (see Figure S4 for the UV–vis spectra and photos). The AuBr_4_^–^ is more stable and difficult to reduce relative to AuCl_4_^–^ because the standard reduction potential of AuBr_4_^–^/Au (0.85 V) is lower than that of AuCl_4_^–^/Au (1.00 V). By solely adjusting the injection rate, two different growth patterns were achieved, corresponding to the situations of twin proliferation and prolongation, respectively. At a slow injection rate of 2 mL h^–1^, a limited number of Au atoms were available, and thus asymmetric axial growth took place, leading to the proliferation of the decahedral twins into Pd–Au Janus icosahedra. Alternatively, at one-shot injection, the solution immediately turned purple, indicating a fast reduction rate. The large number of Au atoms at the initial stage forced symmetric growth along high surface energy sites, resulting in the prolongation of the decahedral twins into Pd@Au core–shell starfishes with a concave surface.

**Figure 2:**
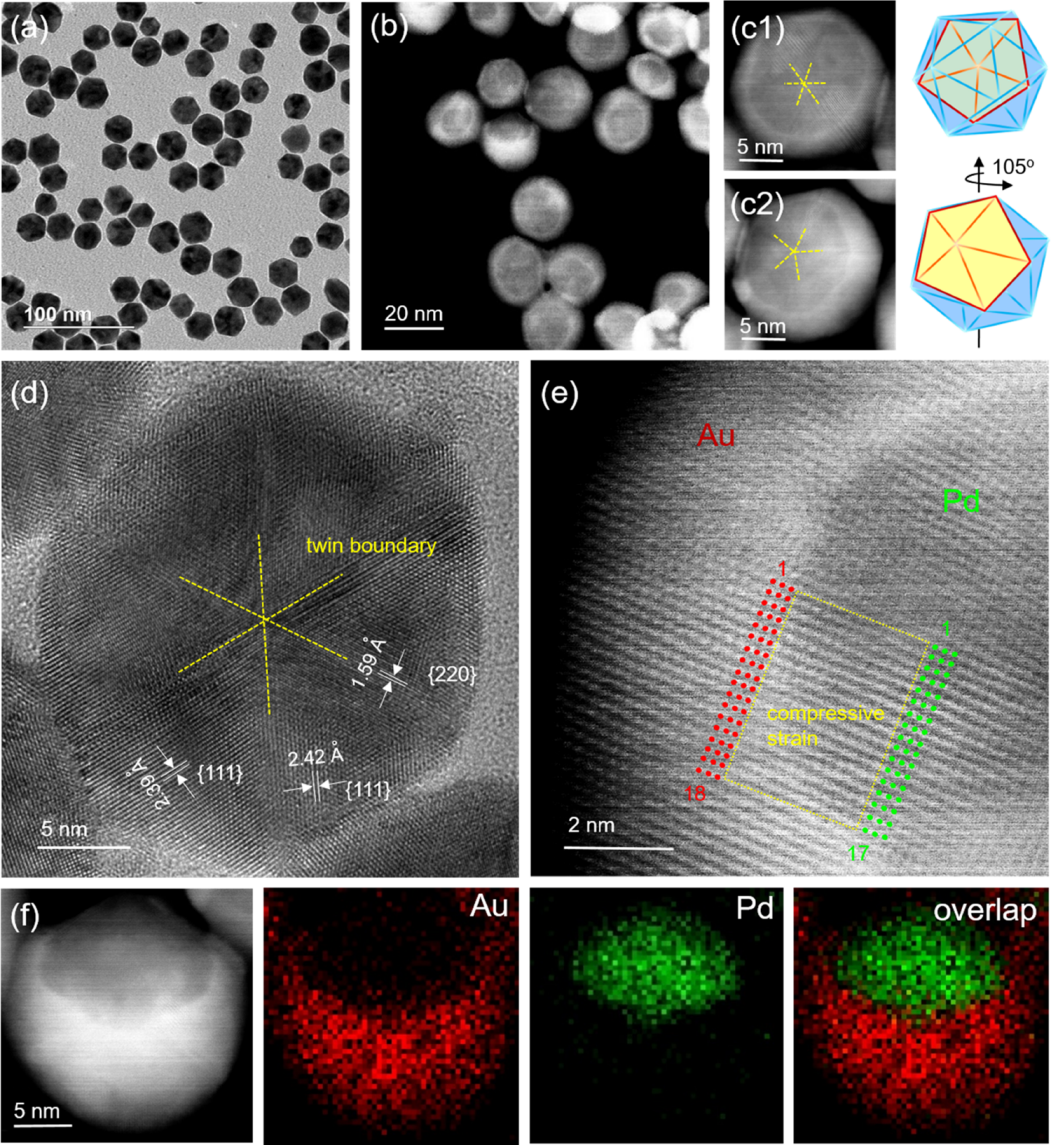
Structural characterizations of the Pd–Au Janus icosahedra. (a) Low-magnification TEM image. (b) HAADF-STEM image. (c) Detailed HAADF-STEM images of individual nanocrystals at two different orientations, together with the corresponding models. (d) HRTEM image viewed along the 6-fold axis. (e) Atomic-resolution HAADF-STEM image showing the compressive strain at the heterometallic junctions. (f) EDX mapping.

Figure 2a shows a representative TEM image of the Pd–Au Janus icosahedra, indicating the formation of regular icosahedra featuring a hexagonal projection and twin boundaries. The average size was determined by measuring the dimension indicated in Figure S2b, and it was calculated to be *ca.* 20.0 ± 1.5 nm. The HAADF-STEM image shows obvious dark contrast between Au and Pd atoms, confirming the asymmetric distribution of the two metals inside the icosahedra (Figure 2b). Importantly, it could be clearly observed that the original Pd decahedral seeds are well preserved in the heterogeneous icosahedra. HAADF-STEM images taken at different angles, alongside their corresponding models, demonstrate the 5- and 6-fold axes inside the icosahedra (Figure 2c), confirming the successful twin proliferation from decahedra into icosahedra. Figure 2d shows a close look at the 6-fold symmetry of the Pd–Au Janus icosahedra. The lattice spacings at faces and axes were measured to be 0.242 and 0.159 nm, corresponding to the {111} and {220} planes, respectively. The HAADF-STEM image in Figure 2e shows the difference in the number of atoms at the Au/Pd boundary region, revealing the creation of compressive strain due to the large lattice mismatch. This mismatch forces the Au atoms next to the Pd decahedron to adapt a compressed atomic arrangement to satisfy the crystal structure. The Janus distribution is further confirmed by EDX mapping. As shown in Figure 2f, the lack of mixing between the green (Pd) and red (Au) regions in the overlap image supports the proposed asymmetric growth mode. The darker Pd decahedral seed at the top of the icosahedral particle corresponds well with the green (Pd) elemental mapping, indicating the complete exposure of the five Pd faces on the surface of each icosahedron. Considering that a typical icosahedron is composed of 20 tetrahedral subunits, the surface of a Pd–Au Janus icosahedron is determined to contain 15 tetrahedral subunits made of Au and five tetrahedral subunits composed of Pd. We then demonstrated the electrocatalytic ORR performances of the Pd–Au Janus icosahedra, using the rotating disk electrode (RDE) in O_2_-saturated 0.1 M KOH solutions. From the positive-going ORR polarization curves shown in Figure S5, the onset and half-wave potentials of Pd–Au Janus icosahedra were measured to be 1.05 and 0.93 V, respectively, which were much more positive than those of the commercial Pd/C catalyst (0.94 and 0.83 V). These results reveal that the Pd–Au Janus icosahedra possess foreseeable utility for electrocatalysis.

**Figure 3:**
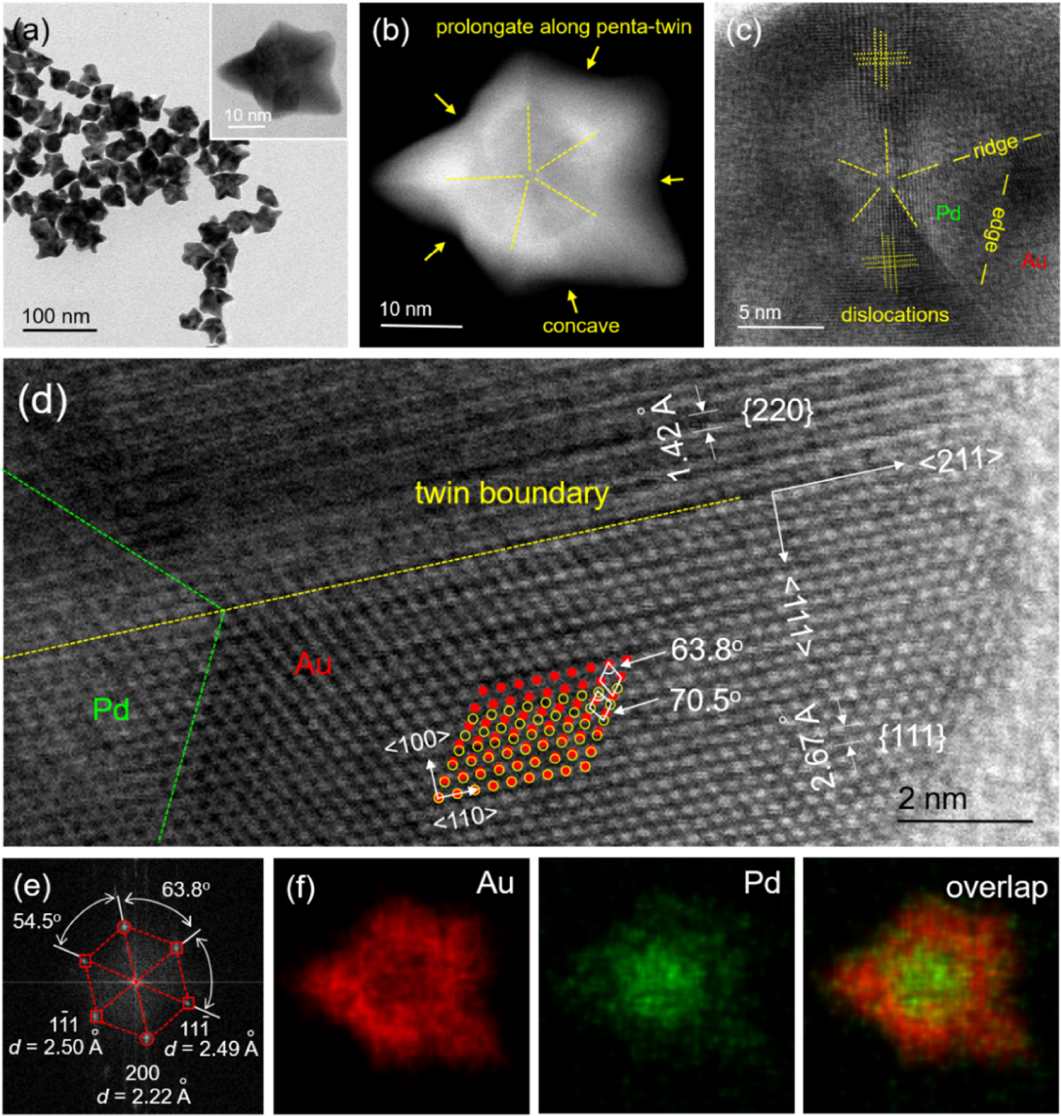
Structural characterizations of the Pd@Au core–shell starfishes. (a) TEM image. (b) HAADF-STEM image highlighting the 5-fold symmetry and the re-entrant grooves. (c, d) HRTEM images showing the dislocations at the heterometallic junctions and the lattice distortion of Au. (e) FFT pattern corresponding to (c). (f) EDX mapping.

Figure 3a shows the TEM image of the Pd@Au core–shell starfishes, with Au growing along the five twin boundaries of each Pd decahedral seed. The starfishes display a star-shaped profile with an average size of 26.5 ± 1.5 nm. The HADDF-STEM image in Figure 3b confirms the core–shell structure, with a Pd decahedron inside each particle. The yellow lines mark the 5-fold twin boundaries, which originate from the center and elongate to generate re-entrant grooves between adjacent twin boundaries. This result implies that Au was deposited preferentially along five ⟨211⟩ ridges during particle growth to form a core–shell structure with anisotropic, concave, and penta-twinned features. Up till now, there have been only reports on the pentagonal twin boundary elongation from decahedral seeds in monometallic or alloy nanocrystals.^30^ Core–shell starfishes have been limited to growth along the low-index ⟨100⟩ directions. Thus, this work represents the first example of lateral prolongation along five high-indexed ⟨211⟩ ridges to produce Pd@Au core–shell starfishes, resulting in a kinetic product instead of a thermodynamic one. Figure 3c shows the bright-field HRTEM image focused on the Pd core region of the starfish, where the ridges and edges of the original decahedral seed can be clearly resolved. Meanwhile, the dislocations due to internal strain arising from a lattice mismatch and angular deficiency are also apparent and highlighted. The HRTEM image in Figure 3d reveals the continuous lattice fringes extending from the Pd core to the Au shell, suggesting that the Au shell took a conformal epitaxial growth on the Pd seed. The lattice constants were measured to be 0.267 and 0.142 nm, corresponding to the {111} and {220} planes of fcc-Au, respectively. Specifically, as illustrated by the solid red circles, the atomic arrangement in the region between the two elongated twin boundaries exhibits a large displacement, with an interplanar angle θ_111∧111(m)_ = 63.8°, which is 9.5% smaller than that of the theoretical fcc-Au (θ_111∧111(m)_ = 70.5°). The mismatch indicates a large extensive lattice strain in the Pd@Au core–shell starfishes because of the necessity to fill the angle gap. The corresponding FFT pattern (Figure 3e) is indexed along the ⟨110⟩ zone axis of the fcc lattice, with interplanar spacings of *d*_200(m)_ = 2.22 Å. The {200} planes show 8.82% expansion relative to the theoretical value of fcc-Au lattice (*d*_200_ = 2.04 Å). The EDX mapping in Figure 3f further confirms a core–shell structure for the penta-twinned starfishes. Specifically, Pd (green) could only be observed in the interior, whereas Au (red) is distributed throughout the outermost extremities to fill in the shape of the starfish.

### Kinetic Effects on Twin Proliferation and Prolongation

**Figure 4:**
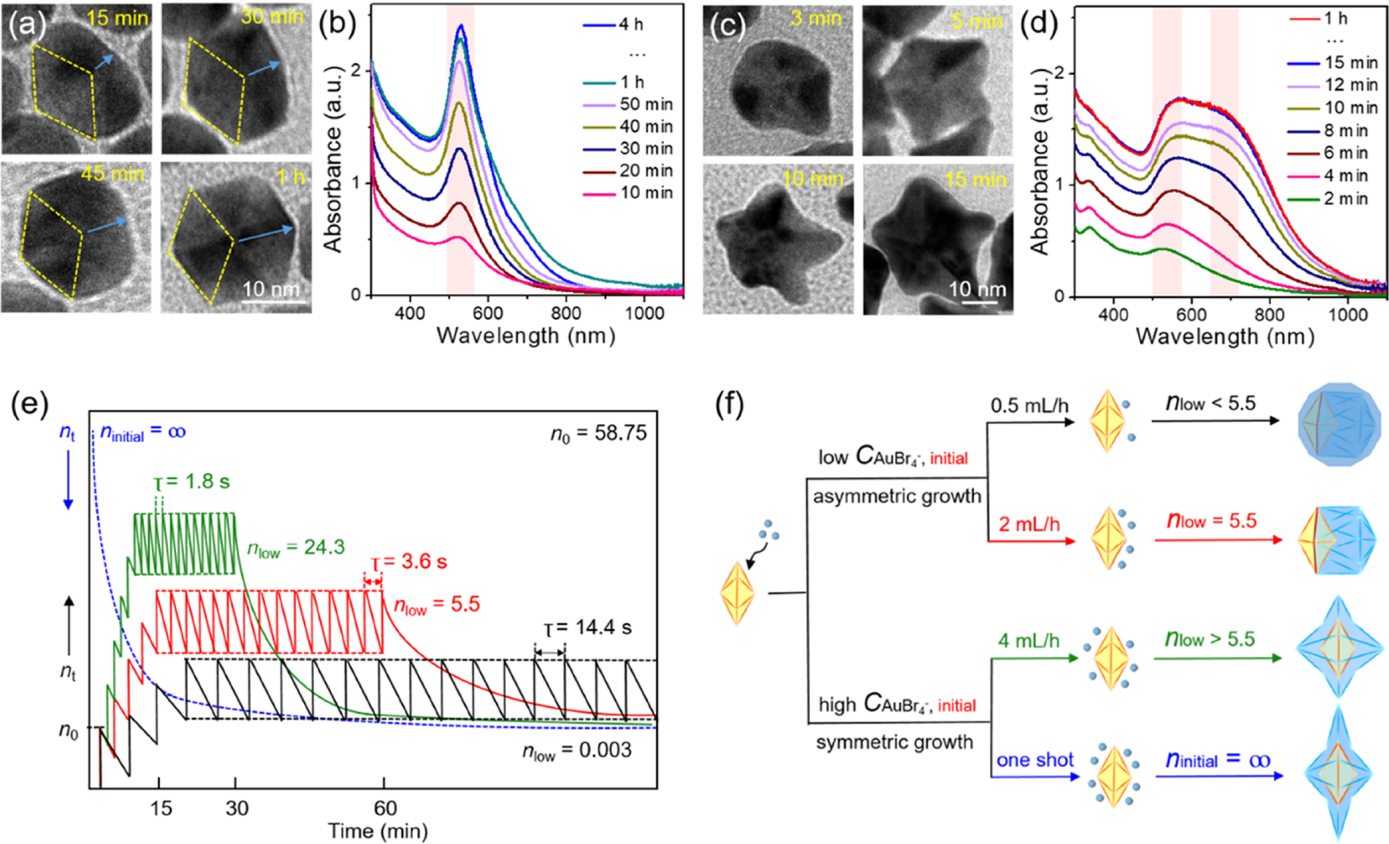
TEM images and UV–vis spectra recorded at different reaction times for (a, b) Pd–Au Janus icosahedra and (c, d) Pd@Au core–shell starfishes. (e) Quantitative analysis showing the plots of instantaneous *n*_0_, τ, *n*_up_, and *n*_low_ in the cases of different injection rates. (f) Schematic showing the dependence of growth pattern on the injection rate of the precursor.

Time-elapsed experiments were conducted to track the growth trajectory of Pd–Au Janus icosahedra (Figures S6 and 4a). The TEM images collected at *t* = 15 min show a pea-like shape with one side of the Pd decahedron exposed on the surface and the other side covered by Au atoms, indicating that the limited supply of Au atoms from the first few drops of precursor could only nucleate on one side of the Pd seed. The TEM images taken at *t* = 30, 45, and 60 min confirm the exclusive deposition of Au atoms on the newly formed Au side with a smaller degree of lattice match relative to the Pd side, generating a Janus icosahedron. The transformation from a decahedron to an icosahedron through the proliferous formation of new twin planes through the asymmetric growth of Au along the axial ⟨110⟩ direction of the Pd seed to lower overall surface free energy is consistent with thermodynamics. The corresponding UV–vis spectra collected at different time points could be used to track the reduction of Au(III) in the system. As shown in Figure 4b, one major absorbance peak at 513 nm emerged at *t* = 10 min, and then the peak intensity became stronger as the injection of Au(III) was continued, making it directly proportional to the increase in particle size.

For the formation of Pd@Au core–shell starfishes, TEM images of the samples collected at *t* = 3, 5, 10, and 15 min display the progressive growth of the multiple-armed pentagram during the reaction, along with an increase in the degree of anisotropy (Figures S7 and 4c). These results indicate that the initial growth began preferentially from vertices, specifically equatorial ones, primarily due to their higher surface free energy originating from the lower atomic coordination number and higher lattice strain. The atoms deposited at these equatorial vertices could then diffuse to either of the two neighboring edges or ridges, while atoms deposited at the two axial vertices could only diffuse along the five ridges. As a result, the majority of the Au atoms accumulated at the ridges, and the diffusion along edges and faces was inadequate, leading to the formation of concave Pd@Au core–shell starfishes with elongated penta-twin boundaries. The corresponding UV–vis spectra collected at different time points showed a major absorbance peak located at 513 nm, which grew in intensity as the reaction was continued and the particle size was increased (Figure 4d). Meanwhile, another peak located at 685 nm began to appear after 8 min into the synthesis, corresponding to the formation of anisotropic structures with transverse and longitudinal modes. These results illustrate the tunability of the surface plasmon resonance of the Pd@Au core–shell starfishes, which exhibited regularity in size, shape, and aspect ratio at different time points.^31^

Since the injection rate directly influences the growth pattern, TEM images acquired at a wide range of injection rates are provided in Figure S8 to show detailed structural changes. As shown in Figure S8a,b, the products obtained at a slow injection rate of 0.5 and 1 mL h^–1^ exhibit irregular polygonal shapes, with the edges becoming rounded, instead of forming regular icosahedra with a hexagonal projection. This result implies that further slowing down the injection rate would decrease the atomic deposition rate while the surface diffusion rate was maintained. As such, the Au atoms could diffuse to the entire surface of the Pd decahedral seed and generate a rounded surface. The products obtained at 3 and 4 mL h^–1^ exhibit a major 5-fold structure with inconspicuous branches, indicating that the symmetric growth slowly becomes predominant under a faster injection rate (Figure S8c,d).

Based on these observations, a quantitative analysis was conducted to understand the relationship between injection rate and growth pattern (see Experimental Section for details).^32^ First, by plotting the instantaneous concentration of the Au(III) precursor remaining in the solution as a function of reaction time, the combined rate constant (*k*) was determined to be 0.682 s^–1^ (Figure S9). By keeping *n*_0_ as a constant and manipulating the τ by tuning the injection rate, *n*_up_ and *n*_low_ could be calculated for different injection rates (as listed in Table S1). Taking a closer look at the quantitative parameters, the key factor responsible for determining the growth pattern was *n*_low_, which was a function of τ only when *n*_0_ was fixed. As shown in Figure 4e, when *n*_low_ ≤ 5.5, the seed-mediated growth of Au on Pd decahedra was exclusively locked in the asymmetric mode. Specifically, the first few drops resulted in a limited number of Au atoms for the deposition on one side of the Pd decahedral seed. The number of Au(III) could then be maintained at a low level by oscillating between *n*_up_ and *n*_low_, leading to slow reduction kinetics and thus generation of Pd–Au Janus icosahedra with symmetry breaking. Otherwise, if *n*_low_ > 5.5, the high concentration of Au(III) quickly pushed the reaction forward at a fast reduction rate. The one-shot injection was an extreme example of this, where the number of Au(III) precursor could reach its maximum almost immediately at n_initial_ and then drastically decreased during the reaction, leading to the symmetric and vertex-preferential growth for the generation of Pd@Au core–shell starfishes (Figure 4f).

It is worth noting that slow surface diffusion rate is an important factor in ensuring the formation of either Pd–Au Janus icosahedra or Pd@Au core–shell starfishes. According to the equation for diffusion coefficient *D*
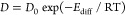
5the slow diffusion rate is closely related to a low reaction temperature. For the synthesis of Pd–Au Janus icosahedra, TEM image of the products acquired at 50 °C displays a polyhedral structure with irregular shape (Figure S10a), which can be attributed to the excessive surface diffusion that disrupted the one-side growth mode. Similarly, in the synthesis of Pd@Au core–shell starfishes, the products obtained at 50 °C display a star-like shape with broadened arms, which could be attributed to additional surface diffusion on edges and faces (Figure S10b). Taken together, it is critical to keep the reaction temperature at 37 °C, which is low enough to ensure slow surface diffusion during the synthesis of both Pd–Au Janus icosahedra and Pd@Au core–shell starfishes.

We also tuned other experiment parameters to investigate the growth mechanism. For the synthesis of Pd–Au Janus icosahedra, when increasing the amount of Au(III) precursor, TEM images of the products still displayed a regular icosahedral shape, along with a larger particle size and rounded corners (Figure S11). Similarly, for the synthesis of Pd@Au core–shell starfishes, TEM images of the products prepared using excess precursor showed multiple-armed pentagrams with obviously lengthened arms (Figure S12). This observation indicates that the dosage of Au(III) did not affect the growth pattern and only influenced the degree of proliferation and elongation. TEM images of the products obtained at different amounts of reducing agent (AA) were also acquired. When the volume of AA was reduced to 20 and 10 μL, there was insufficient growth. The Pd–Au Janus icosahedra displayed an obvious 6-fold twin symmetry with only semi-finished corners (Figure S13), whereas the Pd@Au core–shell starfishes displayed inconspicuous dendritic structures (Figure S14). When the volume of AA was increased to 60 μL, TEM images of the products were almost identical to the products from the standard protocol, indicating that there was a threshold volume of AA needed to drive the formation of the heterometallic structures. Lastly, TEM images of the products obtained in the absence of Ag^+^ ions displayed irregular shapes with anomalous corners and boundaries for both syntheses (Figure S15). This result demonstrates that the Ag^+^ ions played a significant role in guiding shape evolution by acting as an underpotential deposition agent to passivate the surface for Au atom deposition.^33^

### Strain Distributions and Overgrowth Behaviors

**Figure 5:**
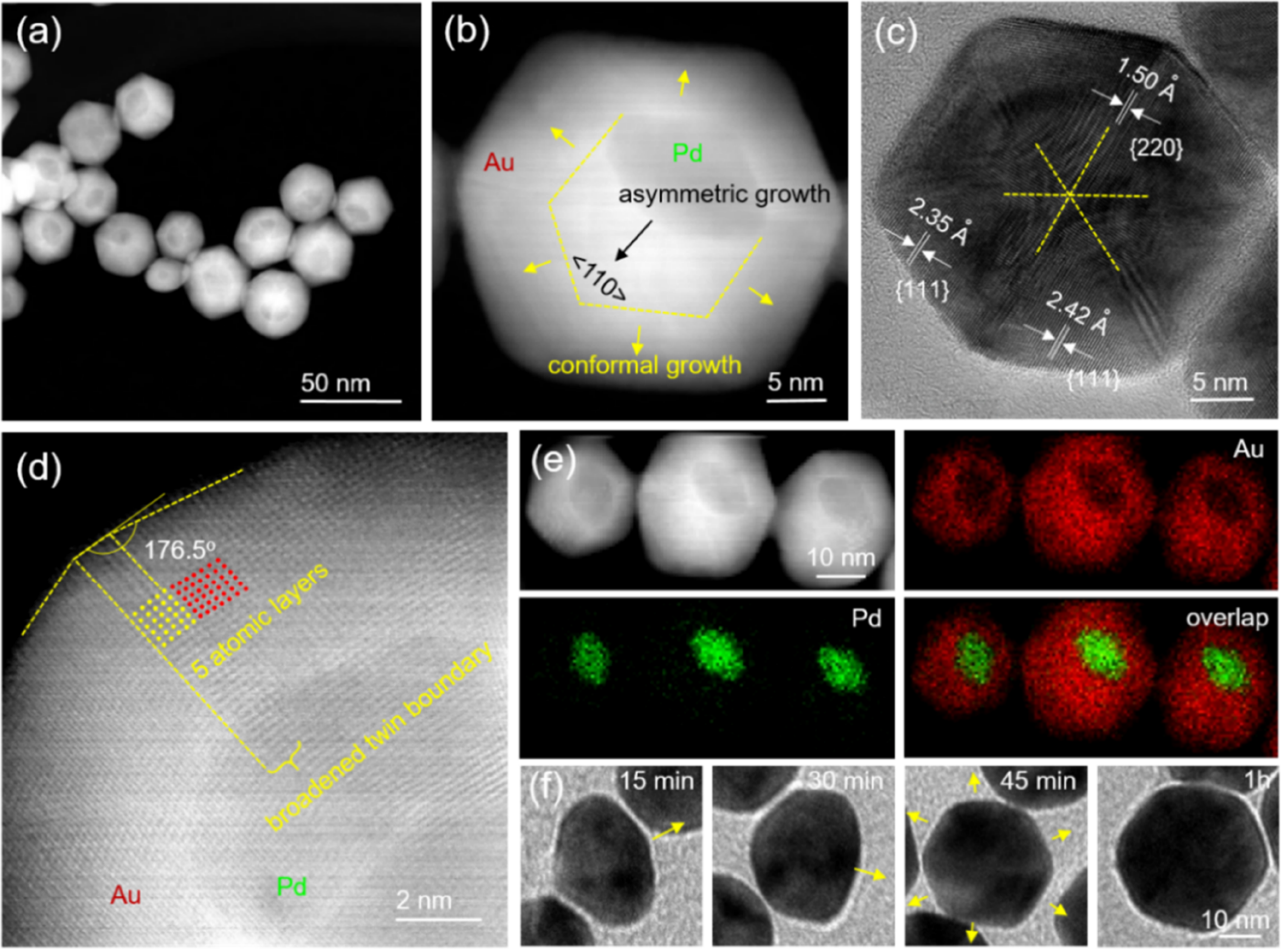
Structural characterizations of the Pd@Au core–shell icosahedra. (a, b) HAADF-STEM images. (c) HRTEM image viewed along the 6-fold axis. (d) Atomic-resolution HAADF-STEM image showing the truncation. (e) EDX mapping images. (f) TEM images at different stages of growth.

The overgrowth behaviors of Au atoms on Pd decahedral seeds were analyzed by reducing the dosage of Pd seed. For the synthesis of Pd–Au Janus icosahedra, TEM images obtained at different seed volumes exhibited an icosahedral shape with tunable sizes of up to 32 nm (Figure S16). Both the size and shape were inversely related to the volume of Pd seeds (Figure S17). However, not all hexagonal profiles and twin boundaries were complete. For larger volumes above 30 μL, the amount of Au atoms per seed was insufficient to form the additional 15 tetrahedral subunits to create a full icosahedron (Figure S16a–c). This resulted in partially completed icosahedra with a shallow depth that could be seen if the particle was sitting on its side. If the volume of Pd seeds was below 30 μL, the amount of Au atoms per decahedral seed was in excess, allowing for the formation of icosahedra along with additional conformal growth that turned them from Janus to core–shell (Figure S16d–f). The Pd@Au core–shell icosahedra with 32 nm in size, corresponding to Figure S16f, were further investigated by HAADF-STEM. The clear contrast between Pd and Au indicates that the Pd seed was no longer situated at the surface of the particle (Figure 5a,b). The yellow outline indicates the limits of the original asymmetric growth along the ⟨110⟩ axis, which then gave way to conformal growth along the directions of the arrows. Figure 5c shows the preservation of the 6-fold symmetry with lattice spacings of 0.242 and 0.150 nm, corresponding to the {111} and {220} planes, respectively. The magnified HAADF-STEM image reveals slight truncation at the vertices, as well as broadened twin boundaries consisting of 5 atomic layers and obtuse junctions (Figure 5d). These surpass the symmetry order of 120° and indicate the split of re-entrant grooves on the sides in order to relax internal crystal strain and reduce surface free energy for the core–shell structure with an enlarged size.^34^ The EDX mapping further confirms the anisotropic core–shell distribution of the Pd@Au icosahedron (Figure 5e). Because the original icosahedra had a Janus structure, with one side of the Pd decahedron exposed, the Pd seed was not located at the center of the final core–shell structure. The TEM images in Figures S18 and 5f validate the asymmetric-conformal growth mechanism as the particles increased in size from 15 to 18, 20, and finally 32 nm, respectively. As depicted by the arrows, it is clear that the growth mode was switched once the icosahedra reached the maximum dimensions of the Pd seed. Afterward, conformal Au deposition led to the thermodynamically favored core–shell structure in order to balance the surface strain.

**Figure 6:**
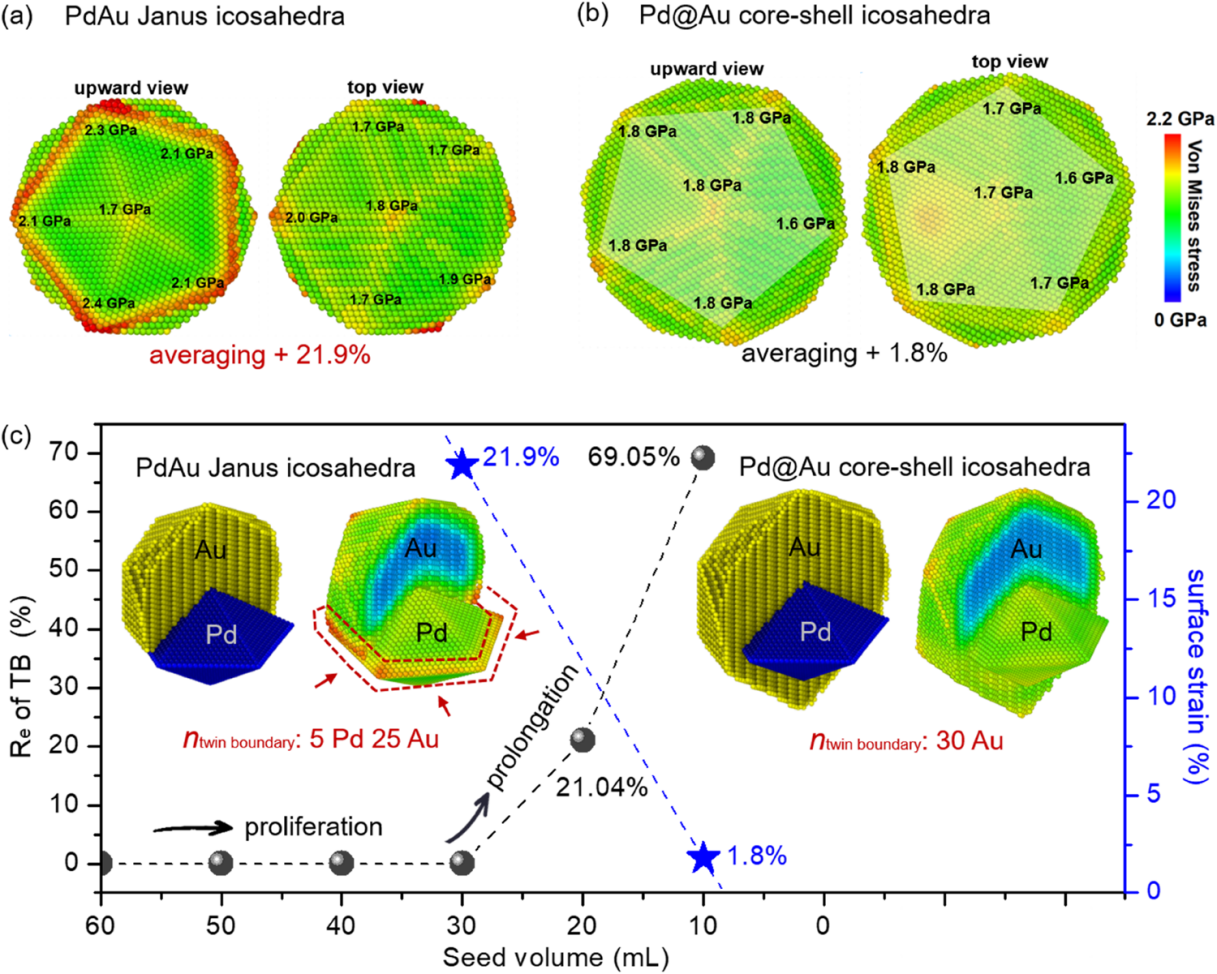
Surface strain fields of a (a) Pd–Au Janus icosahedron and (b) Pd@Au core–shell icosahedron. (c) Summary of the degrees of proliferation and elongation of twin boundary and surface strain difference for Pd–Au Janus icosahedra and Pd@Au core–shell icosahedra, where *n*_twin boundary_ represents the number of twin boundaries exposed on the surface and *R*_e_ represents the elongated ratio of each twin boundary.

We also used MD simulations to analyze the distributions of elastic strain in the heterogeneous Pd–Au icosahedra (Figures 6 and S19). Normally, the surface strain on a pure Au icosahedron is tensile (averaging +0.51%) and symmetrical, with the same strain distribution in each tetrahedral unit.^35^ However, the Pd–Au Janus icosahedra exhibit an asymmetric strain distribution after the relaxation process (Video S1). At the steady state, a strong tensile strain averaging 2.2 GPa is particularly pronounced along the equatorial edges of the Pd decahedra, while the remaining 15 tetrahedral subunits composed of Au show a weak tensile strain (Figure 6a). This leads to a larger surface strain difference averaging up to +21.9% on the surface of the Pd–Au Janus icosahedra. In contrast, the strain distribution fields of the Pd@Au core–shell icosahedra exhibit an equilibrium situation with decreased strain difference (averaging +1.8%), which is possibly due to the weakened effect of internal Pd decahedra (Figure 6b and Video S2). Both the increased size and a larger number of exposed Au atoms tend to take surface reconstruction, thus making the Pd@Au core–shell icosahedra more stable by decreasing the strain difference. The quantitative degrees of proliferation/prolongation of twin boundary in the heterogeneous Pd–Au icosahedra are summarized in Figure 6c. Along with the decrease in seed volume, the number of twin boundaries is proliferating until filling an icosahedron with 30 twin boundaries. At this point, the icosahedron reaches the maximal surface strain difference. Afterward, the 30 twin boundaries start to elongate up to the *R*_e_ of 69.05%, accompanied by the decrease in surface strain difference.

**Figure 7:**
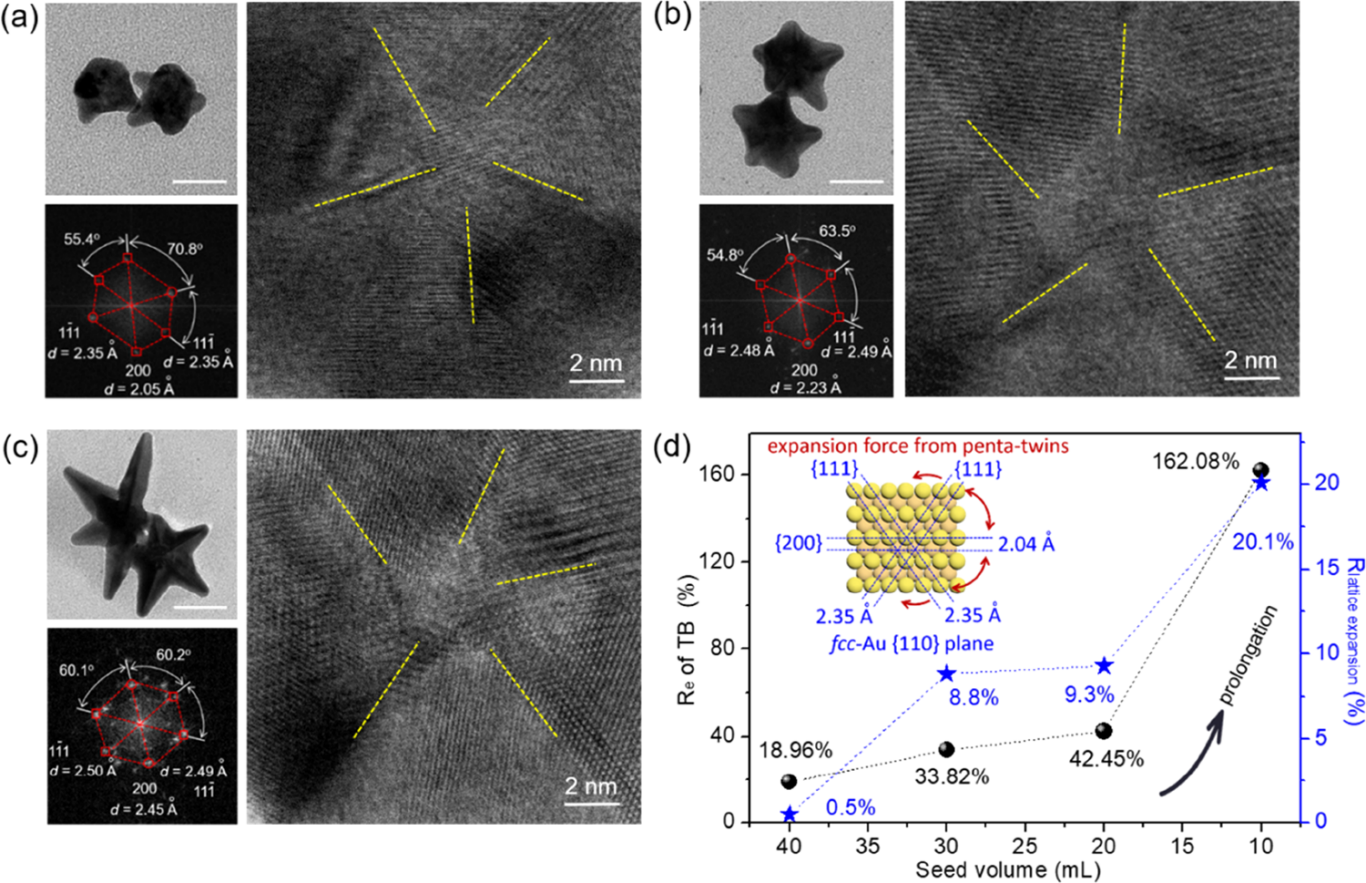
TEM images and corresponding FFT pattern of the Pd@Au core–shell starfishes synthesized using different volumes of Pd decahedral seeds: (a) 40, (b) 20, and (c) 10 μL, respectively. The scale bars in the insets are 20 nm. (d) Summary of the prolongation degree of twin boundary and lattice expansion ratio of Pd@Au core–shell starfishes. *R*_lattice expansion_ represents the lattice expansion ratio of *d*_200(m)_ in comparison with the theoretical value of 2.04 Å.

Similarly, the size, *R*_e_, and lattice expansion ratio of the Pd@Au core–shell starfishes can be controlled by tuning the amount of Pd seeds. As shown in Figures S20 and 7, the size of the Pd@Au core–shell starfishes could be manipulated from 22 to 40 nm by reducing the amount of Pd seeds from 40 to 10 μL, which is again inversely proportional to the amount of Pd seeds added (Figure S21). Likewise, the tip angle of the starfishes could be tuned from 104.2 to 40.1°, while the aspect ratio (*R*_aspect_) increased from 1.84 to 4.12 and the elongated ratio of each twin boundary (*R*_e_) increased from 18.96 to 162.08%, by decreasing the volume of the seeds (listed in Figure S22). With the formation of such highly anisotropic pentagrams, twin interface migration and lattice expansion took place to release luxuriant strain energy. As marked by the yellow lines in Figure 7a–c, the increase of re-entrant grooves causes interface migration of the 5-fold twin boundaries. The corresponding FFT patterns illustrate a tunable interplanar spacing of *d*_200(m)_ ranging from 2.05 to 2.45 Å, along with *d*_111(m)_ ranging from 2.35 to 2.50 Å. The quantitative degrees of prolongation of twin boundary in Pd@Au core–shell starfishes are summarized in Figure 7d. The {200} planes show a tunable expansion of up to 20.10% compared to the theoretical fcc-Au lattice (*d*_200_ = 2.04 Å). This expansion could offer a plasmonic hot spot with large field enhancement and confinement for many tip-enhanced spectroscopies and plasmon-enhanced applications.^36^

## Conclusions

We have systematically investigated twin proliferation and elongation for the fabrication of heterogeneous Pd–Au Janus icosahedra and Pd@Au core–shell starfishes. When the Au(III) precursor is added in one shot or dropwise under *n*_low_ > 5.5, the relatively fast reduction rate promotes symmetric and lateral growth of Au atoms along five high-indexed ⟨211⟩ ridges of Pd decahedral seeds for the generation of concave Pd@Au core–shell starfishes with tunable sizes, twin elongation ratios, and lattice expansion ratios. In the case of dropwise addition and *n*_low_ ≤ 5.5, the slow reduction rate induces asymmetric and axial growth of Au atoms along the ⟨110⟩ orientation of Pd decahedral seeds, producing Pd–Au Janus icosahedra with five Pd faces and 15 Au faces. Such a heterogeneous distribution of elements and twin defects leads to pronounced tensile strain (2.2 GPa) and high strain difference up to +21.9%. This work provides not only an effective method to proliferate and elongate the high-energy twin boundaries but also a deeper understanding of the twin growth kinetics and elastic strain distributions in heterogeneous metal nanocrystals.

## Supporting Information Available

Pd–Au Janus icosahedra exhibit an asymmetric strain distribution after the relaxation process (AVI)
Effect of internal Pd decahedra (AVI)
Additional TEM images, UV–vis data, ORR data, mathematical analysis of the reduction kinetics, MD data, and structural parameter analysis of the samples (PDF)

## Supplementary Material

ja3c03682_si_001.avi

ja3c03682_si_002.avi

## References

[ref1] Huang W.; Johnston-Peck A.; Wolter T.; Chang W.; Yang D.; Xu L.; Oh J.; Reeves B.; Zhou C.; Holtz M.; Herzing A.; Lindenberg A.; Mavrikakis M.; Cargnello M. Steam-Created Grain Boundaries for Methane C–H Activation in Palladium Catalysts. Science 2021, 373, 1518–1523. 10.1126/science.abj5291. 34554810

[ref2] Majee R.; Kumar A.; Das T.; Chakraborty S.; Bhattacharyya S. Tweaking Nickel with Minimal Silver in a Heterogeneous Alloy of Decahedral Geometry to Deliver Platinum-like Hydrogen Evolution Activity. Angew. Chem., Int. Ed. 2020, 59, 2881–2889. 10.1002/anie.201913704. 31825552

[ref3] Wang K.; Wang L.; Yao Z.; Zhang L.; Zhang L.; Yang X.; Li Y.; Wang Y.; Li Y.; Yang F. Kinetic Diffusion-Controlled Synthesis of Twinned Intermetallic Nanocrystals for CO-Resistant Catalysis. Sci. Adv. 2022, 8, eabo4599 10.1126/sciadv.abo4599. 35731880 PMC9217091

[ref4] Zhang X.; Gallagher R.; He D.; Chen G. pH Regulated Synthesis of Monodisperse Penta-Twinned Gold Nanoparticles with High Yield. Chem. Mater. 2020, 32, 5626–5633. 10.1021/acs.chemmater.0c01090.

[ref5] Chen S.; Zhao J.; Su H.; Li H.; Wang H.; Hu Z.; Bao J.; Zeng J. Pd–Pt Tesseracts for the Oxygen Reduction Reaction. J. Am. Chem. Soc. 2021, 143, 496–503. 10.1021/jacs.0c12282. 33386056

[ref6] Li M.; Zhao Z.; Xia Z.; Luo M.; Zhang Q.; Qin Y.; Tao L.; Yin K.; Chao Y.; Gu L.; Yang W.; Yu Y.; Lu G.; Guo S. Exclusive Strain Effect Boosts Overall Water Splitting in PdCu/Ir Core/Shell Nanocrystals. Angew. Chem., Int. Ed. 2021, 60, 8243–8250. 10.1002/anie.202016199. 33434387

[ref7] Lin M.; Wang J.; Kim G.; Liu J.; Pan L.; Lee Y.; Oh J.; Jung Y.; Seo S.; Son Y.; Lim J.; Park J.; Hyeon T.; Nam J. One-Pot Heterointerfacial Metamorphosis for Synthesis and Control of Widely Varying Heterostructured Nanoparticles. J. Am. Chem. Soc. 2021, 143, 3383–3392. 10.1021/jacs.0c11557. 33439007

[ref8] Xie C.; Niu Z.; Kim D.; Li M.; Yang P. Surface and Interface Control in Nanoparticle Catalysis. Chem. Rev. 2020, 120, 1184–1249. 10.1021/acs.chemrev.9b00220. 31580651

[ref9] Zhu R.; Feng H.; Li Q.; Su L.; Fu Q.; Li J.; Song J.; Yang H. Asymmetric Core-Shell Gold Nanoparticles and Controllable Assemblies for SERS Ratiometric Detection of MicroRNA. Angew. Chem., Int. Ed. 2021, 60, 12560–12568. 10.1002/anie.202102893. 33769682

[ref10] Lyu Z.; Zhu S.; Xu L.; Chen Z.; Zhang Y.; Xie M.; Li T.; Zhou S.; Liu J.; Chi M.; Shao M.; Mavrikakis M.; Xia Y. Kinetically Controlled Synthesis of Pd-Cu Janus Nanocrystals with Enriched Surface Structures and Enhanced Catalytic Activities toward CO_2_ Reduction. J. Am. Chem. Soc. 2021, 143, 149–162. 10.1021/jacs.0c05408. 33370094

[ref11] Liu M.; Lyu Z.; Zhang Y.; Chen R.; Xie M.; Xia Y. Twin-Directed Deposition of Pt on Pd Icosahedral Nanocrystals for Catalysts with Enhanced Activity and Durability toward Oxygen Reduction. Nano Lett. 2021, 21, 2248–2254. 10.1021/acs.nanolett.1c00007. 33599510

[ref12] Zhou S.; Zhao M.; Yang T.-H.; Xia Y. Decahedral Nanocrystals of Noble Metals: Synthesis, Characterization, and Applications. Mater. Today 2019, 22, 108–131. 10.1016/j.mattod.2018.04.003.

[ref13] Ruditskiy A.; Vara M.; Huang H.; Xia Y. Oxidative Etching of Pd Decahedral Nanocrystals with a Penta-twinned Structure and Its Impact on Their Growth Behavior. Chem. Mater. 2017, 29, 5394–5400. 10.1021/acs.chemmater.7b01776.

[ref14] Huo D.; Kim M. J.; Lyu Z.; Shi Y.; Wiley B. J.; Xia Y. One-Dimensional Metal Nanostructures: From Colloidal Syntheses to Applications. Chem. Rev. 2019, 119, 8972–9073. 10.1021/acs.chemrev.8b00745. 30854849

[ref15] Zhou L.; Lyu Z.; Xia Y. Pencil-like Ag Nanorods Asymmetrically Capped by Pd. Chem. Mater. 2020, 32, 5361–5367. 10.1021/acs.chemmater.0c01837.

[ref16] Weng W.-L.; Chen H.; Ting Y.; Chen H.; Wu W.; Tu K.; Liao C. Twin-Boundary Reduced Surface Diffusion on Electrically Stressed Copper Nanowires. Nano Lett. 2022, 22, 9071–9076. 10.1021/acs.nanolett.2c03437. 36342418

[ref17] Xu L.; Wang K.; Jiang B.; Chen W.; Liu F.; Hao H.; Zou C.; Yang Y.; Huang S. Competitive Effect in The Growth of Pd-Au-Pd Segmental Nanorods. Chem. Mater. 2016, 28, 7394–7403. 10.1021/acs.chemmater.6b02871.

[ref18] Wang X.; Choi S.; Roling L.; Luo M.; Ma C.; Zhang L.; Chi M.; Liu J.; Xie Z.; Herron J.; Mavrikakis M.; Xia Y. Palladium-Platinum Core-Shell Icosahedra with Substantially Enhanced Activity and Durability towards Oxygen Reduction. Nat Commun. 2015, 6, 7594 10.1038/ncomms8594. 26133469 PMC4506534

[ref19] Bian T.; Zhang H.; Jiang Y.; Jin C.; Wu J.; Yang H.; Yang D. Epitaxial Growth of Twinned Au-Pt Core-Shell Star-Shaped Decahedra as Highly Durable Electrocatalysts. Nano Lett. 2015, 15, 7808–7815. 10.1021/acs.nanolett.5b02960. 26524225

[ref20] Wang Y.; He J.; Liu C.; Chong W. H.; Chen H. Thermodynamics versus Kinetics in Nanosynthesis. Angew. Chem., Int. Ed. 2015, 54, 2022–2051. 10.1002/anie.201402986. 25536948

[ref21] Ruditskiy A.; Zhao M.; Gilroy K.; Vara M.; Xia Y. Toward a Quantitative Understanding of the Sulfate-Mediated Synthesis of Pd Decahedral Nanocrystals with High Conversion and Morphology Yields. Chem. Mater. 2016, 28, 8800–8806. 10.1021/acs.chemmater.6b04528.

[ref22] Luo M.; Huang H.; Choi S.; Zhang C.; Silva R.; Peng H.; Li Z.; Liu J.; He Z.; Xia Y. Facile Synthesis of Ag Nanorods with No Plasmon Resonance Peak in the Visible Region by Using Pd Decahedra of 16 nm in Size as Seeds. ACS Nano 2015, 9, 10523–10532. 10.1021/acsnano.5b05053. 26372854

[ref23] Wang C.; Huang Z.; Ding Y.; Xie M.; Chi M.; Xia Y. Facet-Controlled Synthesis of Platinum-Group-Metal Quaternary Alloys: The Case of Nanocubes and {100} Facets. J. Am. Chem. Soc. 2023, 145, 2553–2560. 10.1021/jacs.2c12368. 36576951

[ref24] Zhou M.; Wang H.; Vara M.; Hood Z.; Luo M.; Yang T.; Bao S.; Chi M.; Xiao P.; Zhang Y.; Xia Y. Quantitative Analysis of the Reduction Kinetics Responsible for the One-Pot Synthesis of Pd-Pt Bimetallic Nanocrystals with Different Structures. J. Am. Chem. Soc. 2016, 138, 12263–12270. 10.1021/jacs.6b07213. 27568848

[ref25] Plimpton S. Fast Parallel Algorithms for Short-Range Molecular Dynamics. J. Comput. Phys. 1995, 117, 1–19. 10.1006/jcph.1995.1039.

[ref26] Wang X.; Vara M.; Luo M.; Huang H.; Ruditskiy A.; Park J.; Bao S.; Liu J.; Howe J.; Chi M.; Xie Z.; Xia Y. Pd@Pt Core-Shell Concave Decahedra: A Class of Catalysts for the Oxygen Reduction Reaction with Enhanced Activity and Durability. J. Am. Chem. Soc. 2015, 137, 15036–15042. 10.1021/jacs.5b10059. 26566188

[ref27] Chen Y.; Huang Q.; Zhao S.; Zhou H.; Wang J. Penta-Twin Destruction by Coordinated Twin Boundary Deformation. Nano Lett. 2021, 21, 8378–8384. 10.1021/acs.nanolett.1c02970. 34591495

[ref28] Ma X.; Lin F.; Chen X.; Jin C. Unveiling Growth Pathways of Multiply Twinned Gold Nanoparticles by In Situ Liquid Cell Transmission Electron Microscopy. ACS Nano 2020, 14, 9594–9604. 10.1021/acsnano.9b10173. 32806061

[ref29] Wang C.; Li X.; Jin L.; Lu P.-H.; Dejoie C.; Zhu W.; Wang Z.; Bi W.; Dunin-Borkowski R. E.; Chen K.; Jin M. Etching-Assisted Route to Heterophase Au Nanowires with Multiple Types of Active Surface Sites for Silane Oxidation. Nano Lett. 2019, 19, 6363–6369. 10.1021/acs.nanolett.9b02532. 31361961

[ref30] Velázquez-Salazar J. J.; Bazán-Díaz L.; Zhang Q.; Mendoza-Cruz R.; Montaño-Priede L.; Guisbiers G.; Large N.; Link S.; José-Yacamán M. Controlled Overgrowth of Five-Fold Concave Nanoparticles into Plasmonic Nanostars and Their Single-Particle Scattering Properties. ACS Nano 2019, 13, 10113–10128. 10.1021/acsnano.9b03084. 31419107

[ref31] Smith J. D.; Bladt E.; Burkhart J.; Winckelmans N.; Koczkur K.; Ashberry H. M.; Bals S.; Skrabalak S. Defect-Directed Growth of Symmetrically Branched Metal Nanocrystals. Angew. Chem., Int. Ed. 2020, 59, 943–950. 10.1002/anie.201913301. 31721406

[ref32] Peng H.-C.; Park J.; Zhang L.; Xia Y. Toward a Quantitative Understanding of Symmetry Reduction Involved in the Seed-Mediated Growth of Pd Nanocrystals. J. Am. Chem. Soc. 2015, 137, 6643–6652. 10.1021/jacs.5b03040. 25941798

[ref33] Tsao Y.-C.; Rej S.; Chiu C.-Y.; Huang M. Aqueous Phase Synthesis of Au-Ag Core-Shell Nanocrystals with Tunable Shapes and Their Optical and Catalytic Properties. J. Am. Chem. Soc. 2014, 136, 396–404. 10.1021/ja410663g. 24341355

[ref34] Gilroy K. D.; Elnabawy A.; Yang T.; Roling L.; Howe J.; Mavrikakis M.; Xia Y. Thermal Stability of Metal Nanocrystals: An Investigation of the Surface and Bulk Reconstructions of Pd Concave Icosahedra. Nano Lett. 2017, 17, 3655–3661. 10.1021/acs.nanolett.7b00844. 28448153

[ref35] Wang L.; Zhao S.; Liu C.; Li C.; Li X.; Li H.; Wang Y.; Ma C.; Li Z.; Zeng J. Aerobic Oxidation of Cyclohexane on Catalysts Based on Twinned and Single-Crystal Au_75_Pd_25_ Bimetallic Nanocrystals. Nano Lett. 2015, 15, 2875–2880. 10.1021/nl5045132. 25839191

[ref36] Rossi T. P.; Erhart P.; Kuisma M. Hot-Carrier Generation in Plasmonic Nanoparticles: The Importance of Atomic Structure. ACS Nano 2020, 14, 9963–9971. 10.1021/acsnano.0c03004. 32687311 PMC7458472

