# Supplementary material for: Twin Proliferation and Prolongation under Kinetic Control: Pd–Au Janus Icosahedra versus Pd@Au Core–Shell Starfishes
Source: J Am Chem Soc. 2023 Jun 9;145(24):13400–10. doi: 10.1021/jacs.3c03682 (PMC10288509; doi:10.1021/jacs.3c03682)
Supplement: Supplementary file 3 — ja3c03682_si_003.pdf [file ja3c03682_si_003.pdf]

Twin Proliferation and Prolongation under Kinetic Control: Pd-Au Janus Icosahedra *versus* Pd@Au Core-shell Starfishes

Xiaoyu Qiu,[†,‡] Veronica Pawlik,[§] Shan Zhou,[§] Jing Tao,[⊥] and Younan Xia[*,†]

[†]The Wallace H. Coulter Department of Biomedical Engineering, Georgia Institute of Technology and Emory University, Atlanta, Georgia 30332, United States

[‡]School of Chemistry and Materials Science, Nanjing Normal University, Nanjing 210023, P. R. China

[§]School of Chemistry and Biochemistry, Georgia Institute of Technology, Atlanta, Georgia 30332, United States

[⊥]Condensed Matter Physics and Materials Science Department, Brookhaven National Laboratory, Upton, New York 11973, United States

[*]Corresponding author. Email: younan.xia@bme.gatech.edu

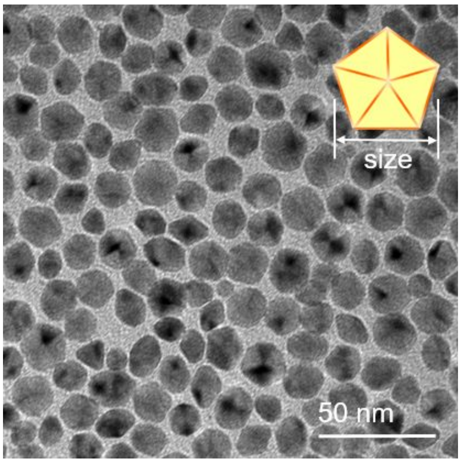

Figure S1. TEM image of the penta-twinned Pd decahedral seeds used for Au growth.

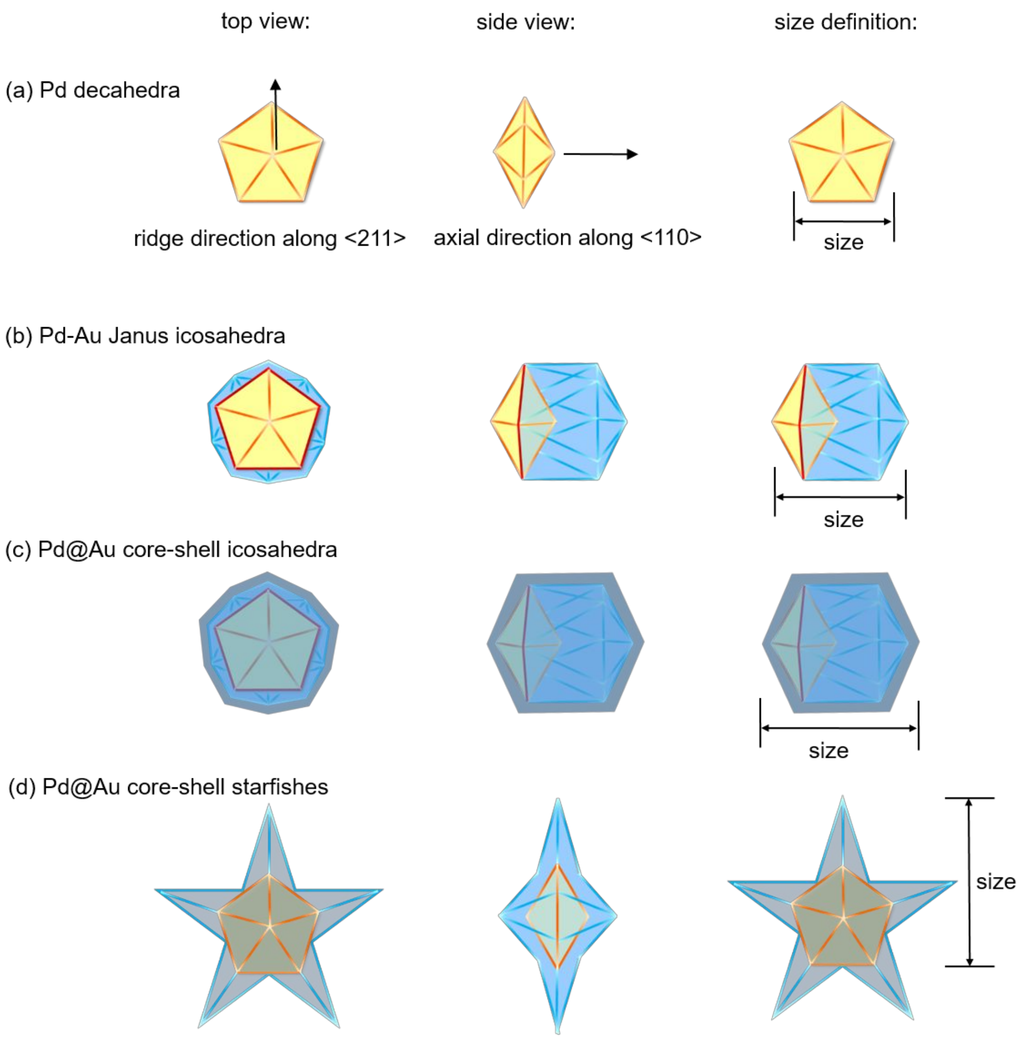

Figure S2. Schematics of the top view, side view, and size definitions of a (a) pure Pd decahedron, (b) Pd-Au Janus icosahedron, (c) Pd@Au core-shell icosahedron, and (d) Pd@Au core-shell starfish.

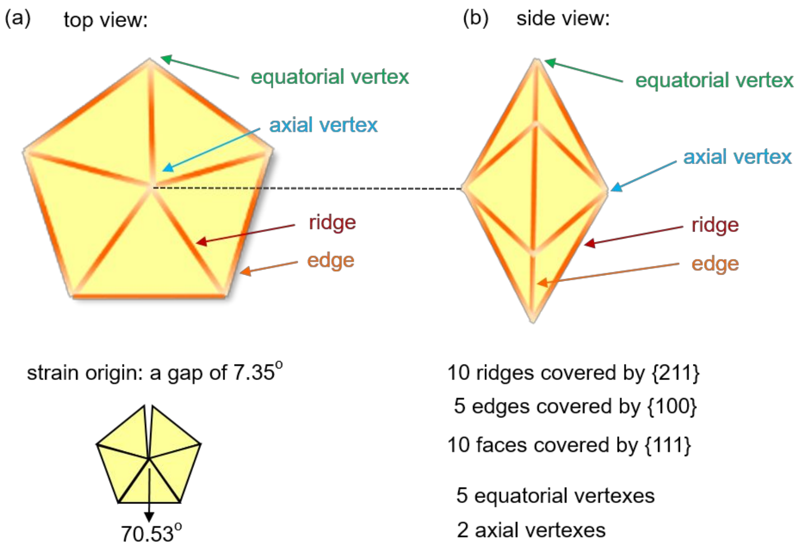

Figure S3. Schematics of the (a) top view and (b) side view of a decahedron, showing the ridges, edges, facets, axial vertices, and equatorial vertices.

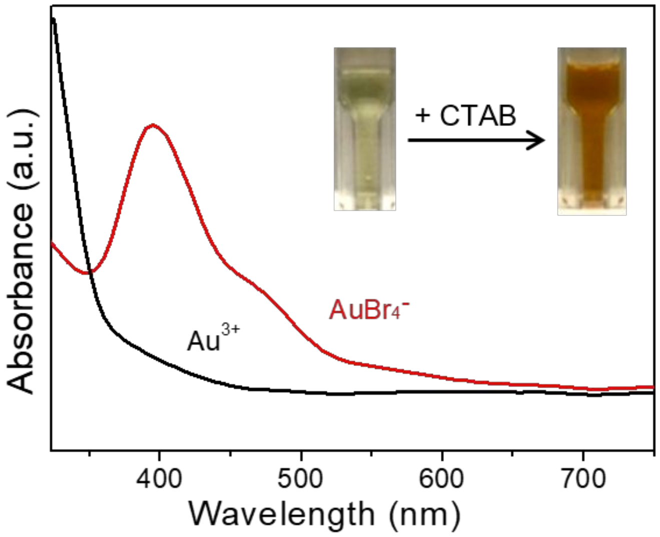

Figure S4. UV-vis absorption spectra of the $HAuCl_4$ solution before and after the introduction of CTAB.

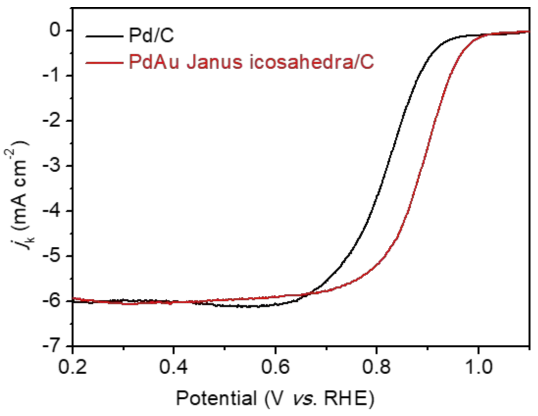

Figure S5. ORR polarization curves of the Pd-Au Janus icosahedra in O_2-saturated 0.1 M KOH solution, scan rate: 5 mV/s and rotation rate: 1600 rpm. The Pd-Au Janus icosahedra were loaded onto a carbon support (Vulcan XC-72) with a metal loading content of 20%.

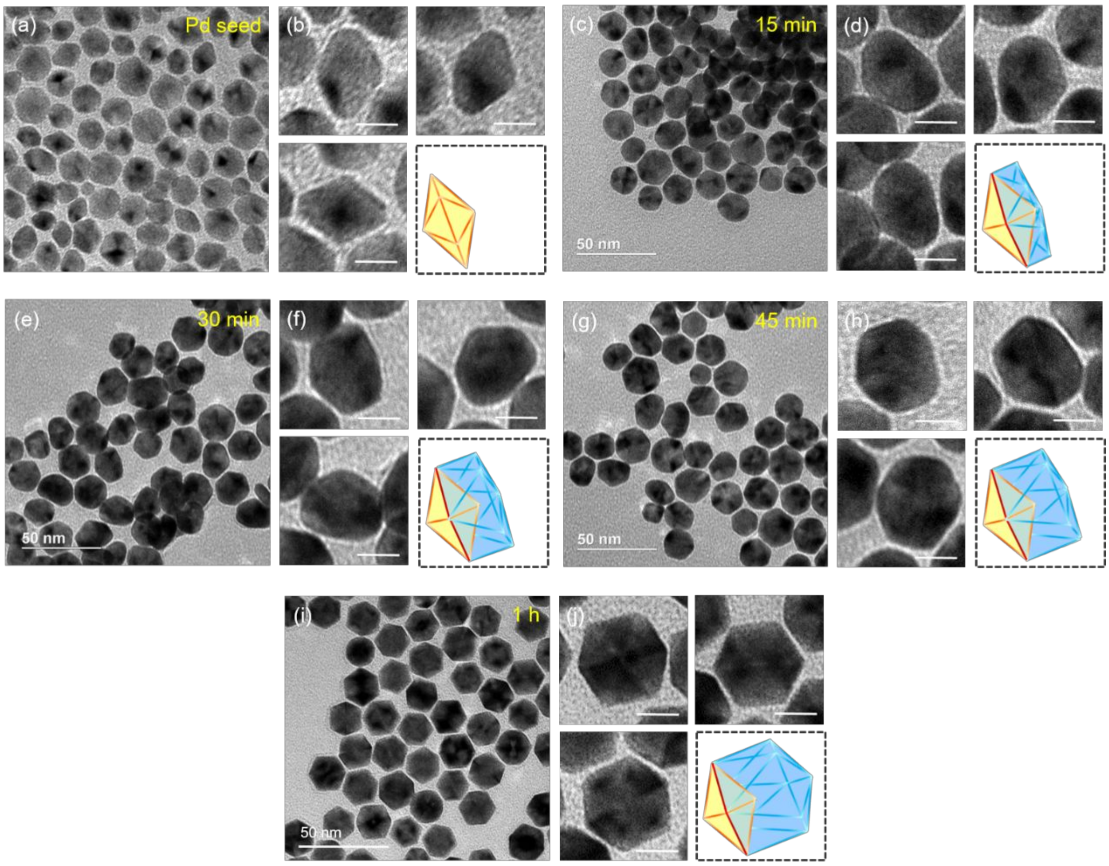

Figure S6. TEM images of the Pd-Au Janus icosahedra prepared using the standard protocol except for the variation in reaction time. The scale bars in the insets are 10 nm.

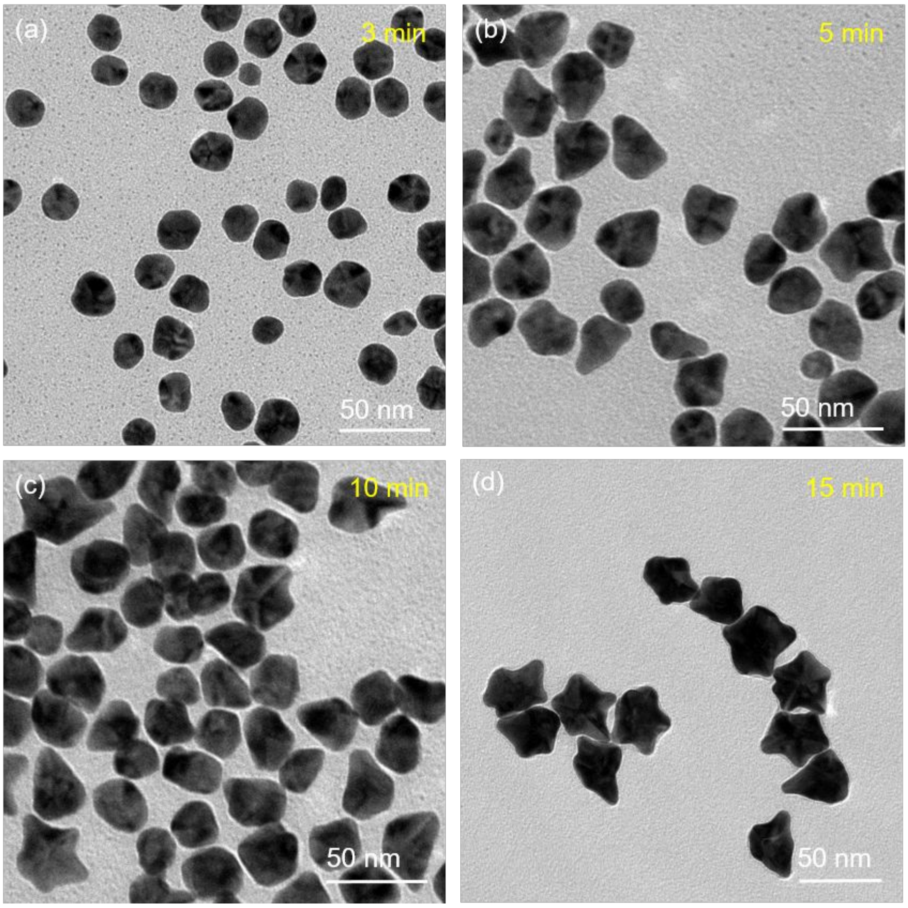

Figure S7. TEM images of the Pd@Au core-shell starfishes prepared using the standard protocol except for the variation in reaction time.

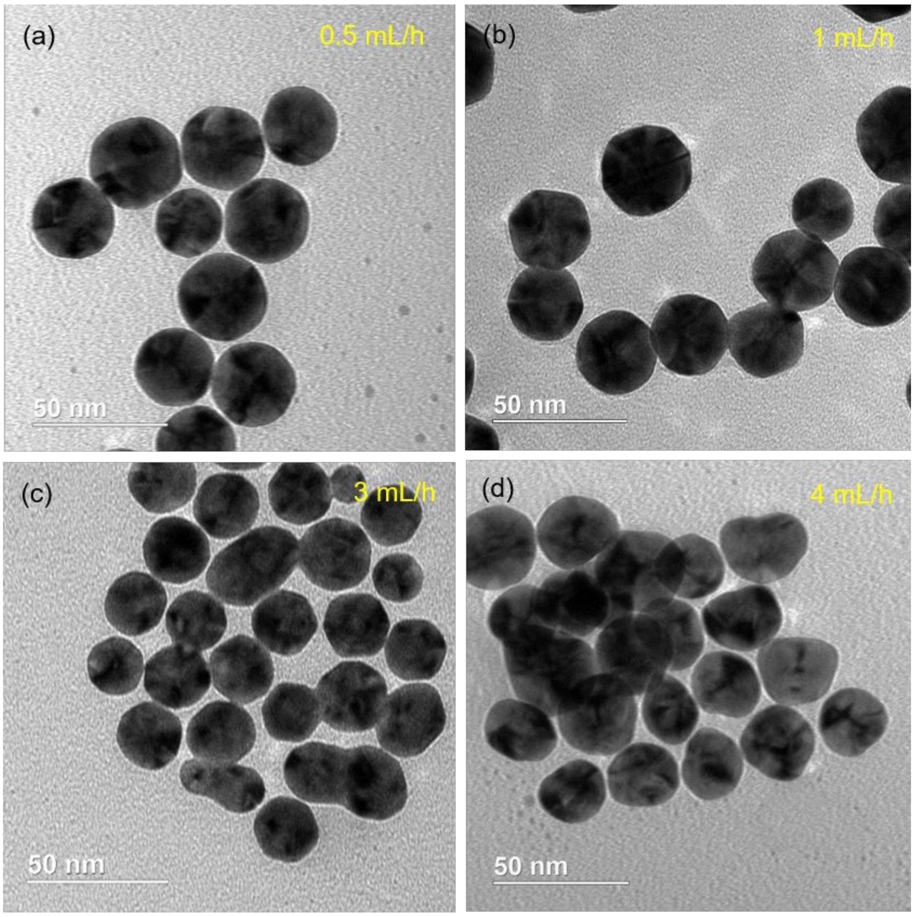

Figure S8. TEM images of the products prepared using the standard protocol except for the variation in injection rate: (a) 0.5, (b) 1, (c) 3, and (d) 4 mL/h.

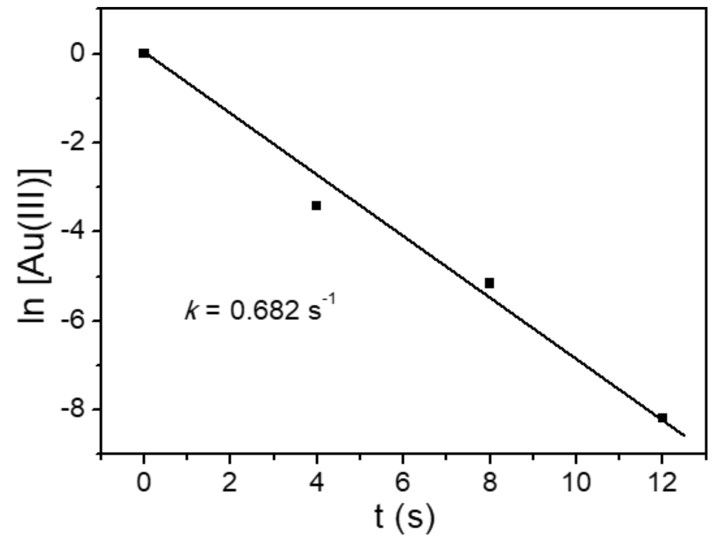

Figure S9. (b) Plot of ln[Au(III)] as a function of reaction time, giving a straight line with a slope that corresponds to -*k*.

Table S1. Summary of the parameters involved in the seed-mediated growth of Au on Pd decahedral seeds at different injection rates.

Injection rate (mL/h)	n_0	τ (s)	n_{up}	n_{low}
0.5	58.75	14.4	58.8	0.003
1	58.75	7.2	59.2	0.436
2	58.75	3.6	64.3	5.5
3	58.75	2.4	72.9	14.2
4	58.75	1.8	83.1	24.3

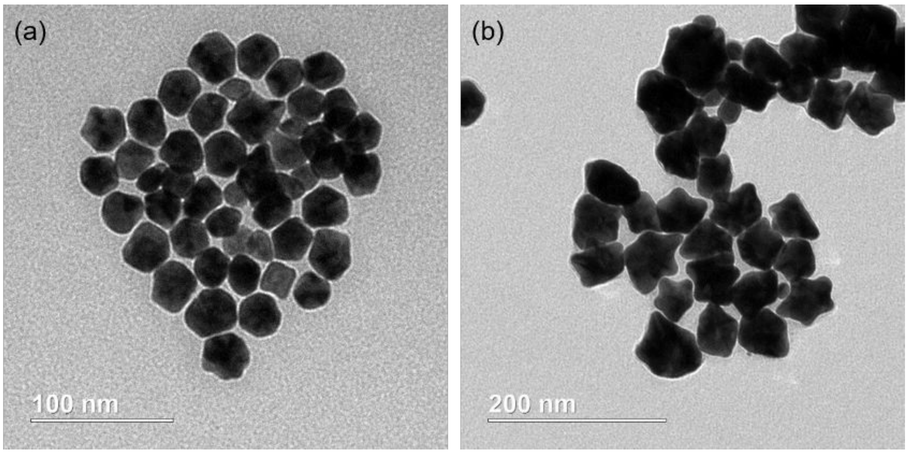

Figure S10. TEM images of the products synthesized using the standard protocol for (a) Pd-Au Janus icosahedra and (b) Pd@Au core-shell starfishes, respectively, except that the reaction temperature was raised to 50 ^{o}C.

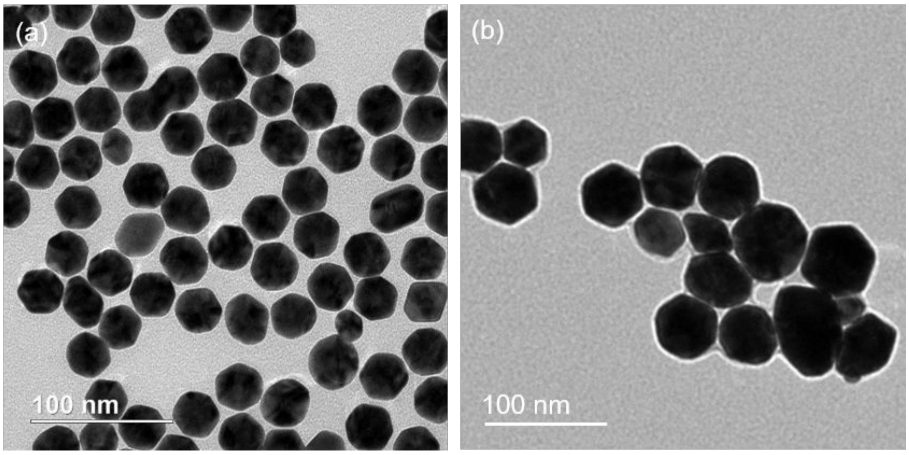

Figure S11. TEM images of the Pd-Au Janus icosahedra synthesized using the standard protocol except for the amount of $HAuCl_4$ solution: (a) 3 and (b) 4 mL.

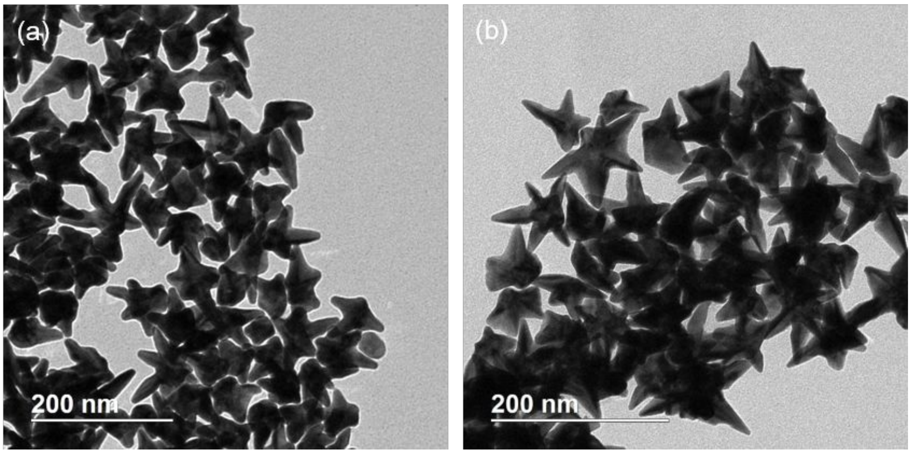

Figure S12. TEM images of the Pd@Au core-shell starfishes synthesized using the standard protocol except for the amount of $HAuCl_4$ solution: (a) 3 and (b) 4 mL.

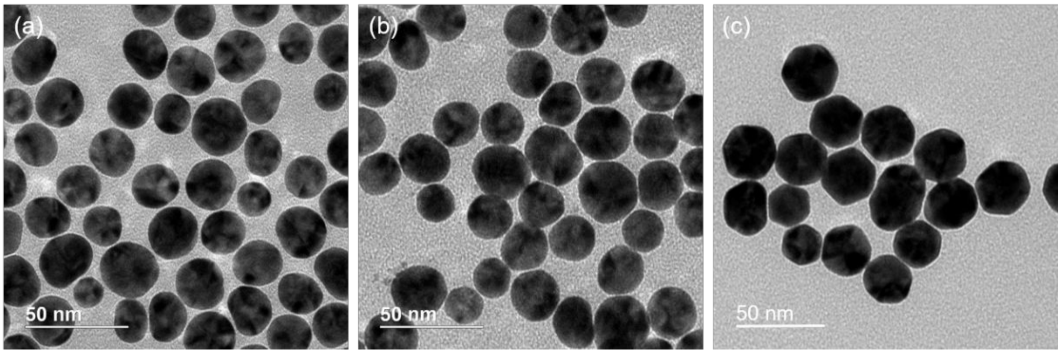

Figure S13. TEM images of the Pd-Au Janus icosahedra prepared using the standard protocol except for the amount of AA: (a) 10, (b) 20, and (c) 60 μL.

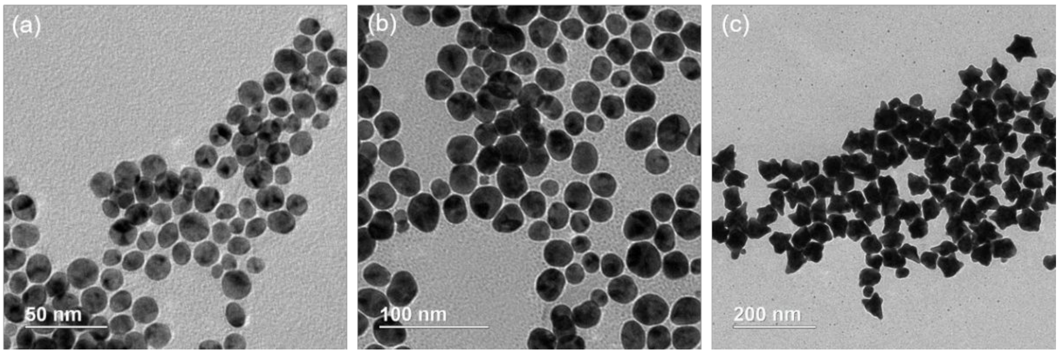

Figure S14. TEM images of the Pd@Au core-shell starfishes synthesized using the standard protocol except for the amount of AA: (a) 10, (b) 20, and (c) 60 μL.

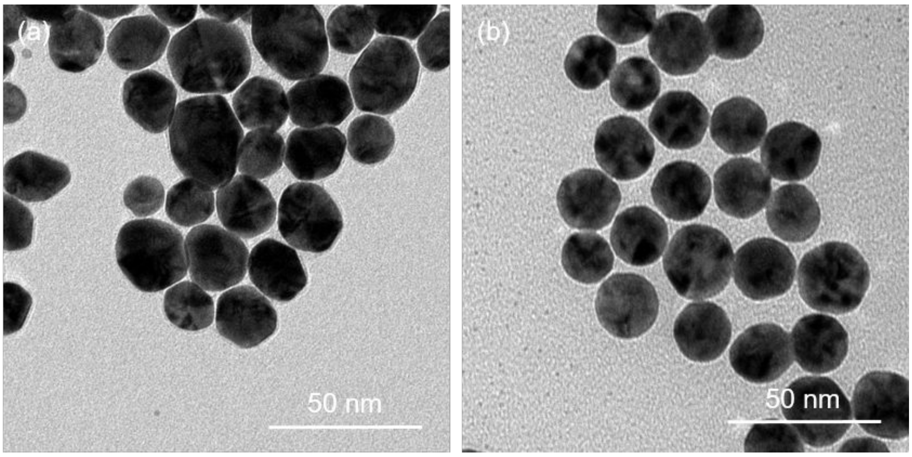

Figure S15. TEM images of the products synthesized using the standard protocol without the involvement of Ag^{+} ions: (a) Pd-Au Janus icosahedra and (b) Pd@Au core-shell starfishes.

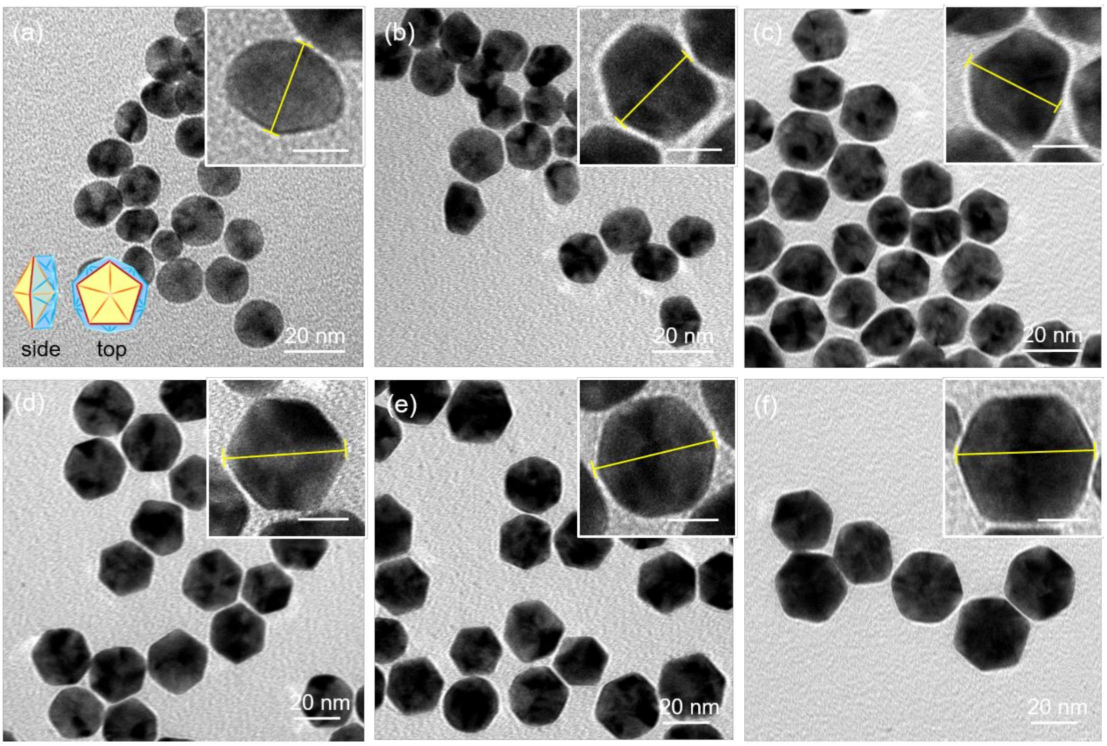

Figure S16. TEM images of the Pd-Au Janus icosahedra prepared using the standard protocol except that different volumes of Pd decahedral seeds were used: (a) 60, (b) 50, (c) 40, (d) 30, (e) 20, and (f) 10 μL, respectively. The scale bars in the insets are 10 nm.

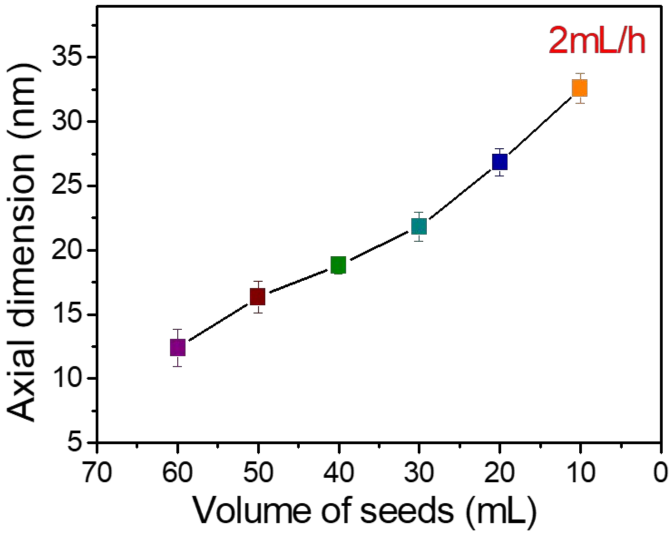

Figure S17. Axial dimension of the Pd-Au Janus icosahedra as a function of the volume of Pd decahedral seeds in the case of 2 mL/h injection rate.

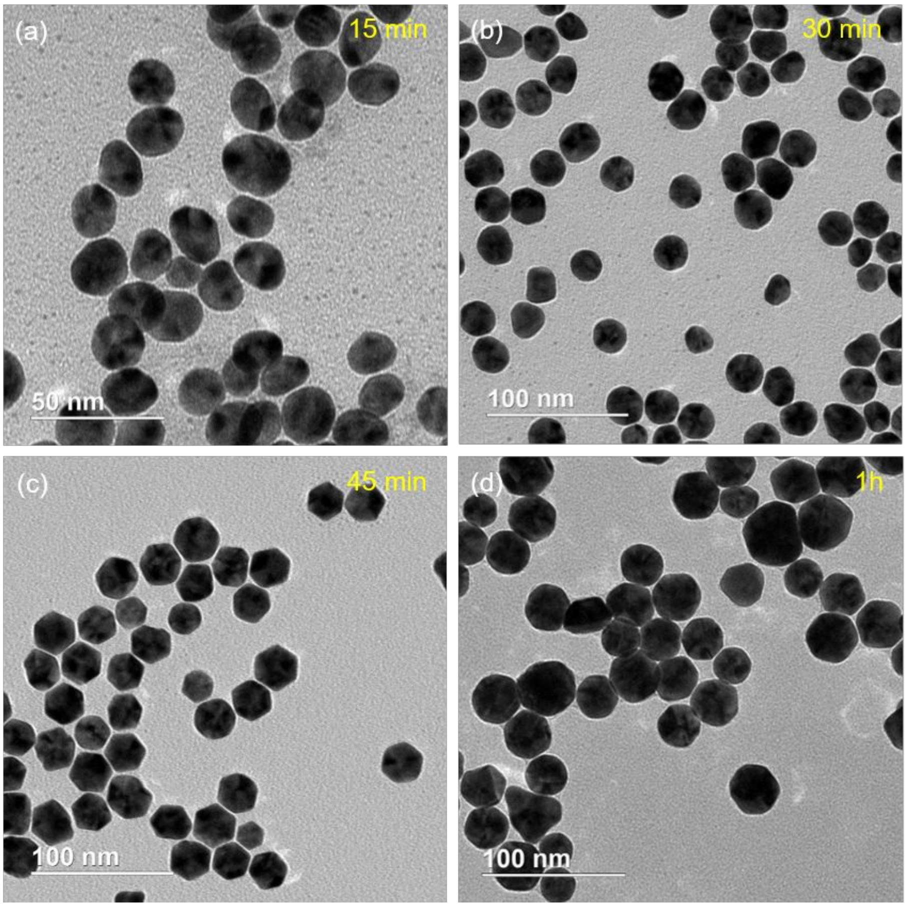

Figure S18. TEM images of the Pd@Au core-shell icosahedra prepared using the standard protocol except for the variation in reaction time.

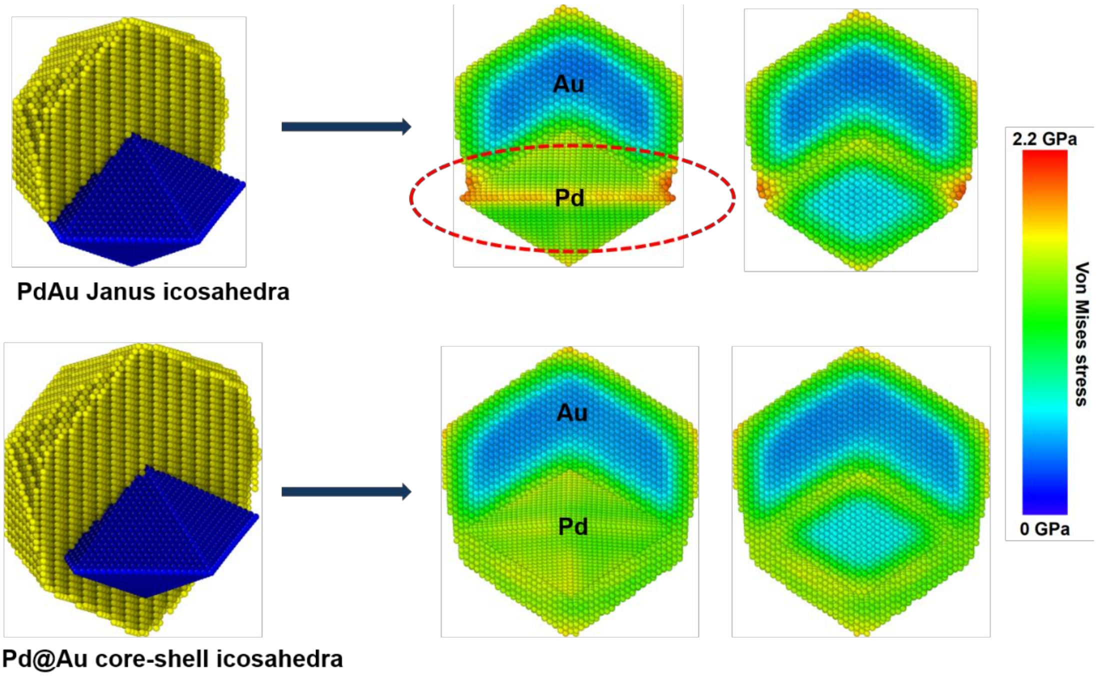

Figure S19. Atomic structures and profiles showing the asymmetric elastic strains in a Pd-Au Janus icosahedron and a Pd@Au core-shell icosahedron, respectively.

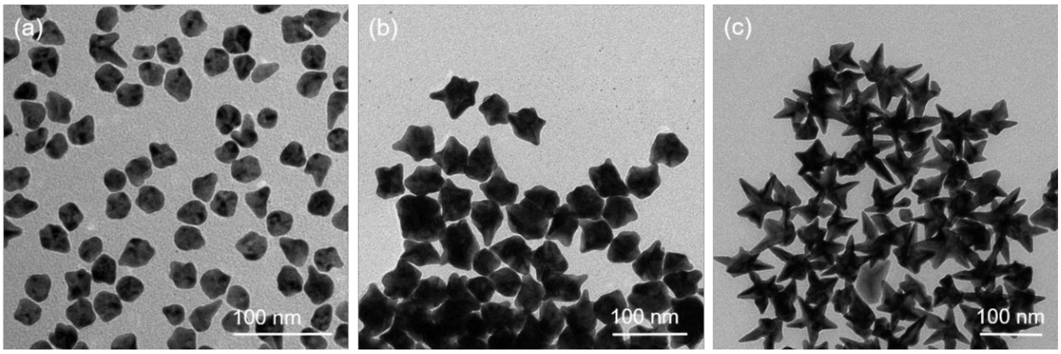

Figure S20. TEM images of the Pd@Au core-shell starfishes synthesized using the standard protocol except that different volumes of Pd decahedral seeds were used: (a) 40, (b) 20, and (c) 10 μL, respectively.

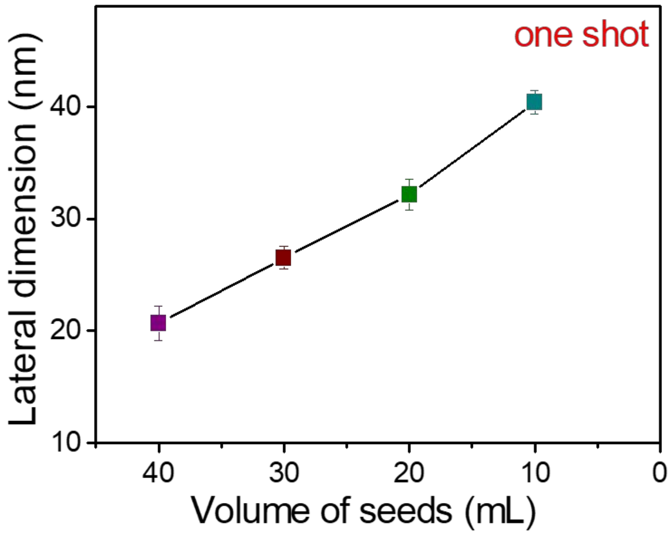

Figure S21. Literal dimension of the Pd@Au core-shell starfishes as a function of the volume of Pd decahedral seeds in the case of one-shot injection.

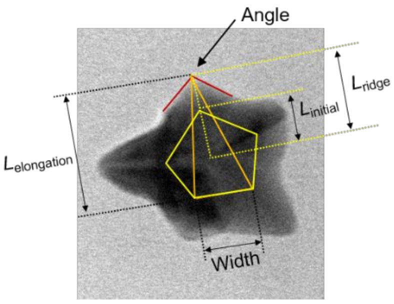

$R_{aspect} = L_{elongation}/\text{Width}$

$R_e = (L_{ridge} - L_{initial}) / (L_{initial})$

Volume of seeds	$\text{Angle}_{average}$	R_{aspect}	R_e
40 μL	104.2°	1.84	18.96
30 μL	99.5°	2.02	33.82
20 μL	86.9°	2.49	42.45
10 μL	40.1°	4.12	162.08

Figure S22. Summary of the detailed structural parameters, together with the corresponding definitions and calculations, of the Pd@Au core-shell starfishes.
